# Gangliosides modulate the secretion of extracellular vesicles and their misfolded protein cargo

**DOI:** 10.1126/sciadv.ady5212

**Published:** 2025-09-17

**Authors:** John Monyror, Vaibhavi Kadam, Luis Carlos Morales, Diego Ordóñez, Jeremies Ibanga, Asifa K. Zaidi, Emily McNamara, Desmond Pink, Magnus Stenlund, Ken Reyes, Aislinn D. Maguire, Jing Huang, Leonardo M. Cortez, Valerie L. Sim, Sue-Ann Mok, Elena Posse de Chaves, Simonetta Sipione

**Affiliations:** ^1^ Neuroscience and Mental Health Institute, University of Alberta, Edmonton, AB T6G2R3, Canada.; ^2^ Glycomics Institute of Alberta, University of Alberta, Edmonton, AB T6G2R3, Canada.; ^3^ Department of Pharmacology, University of Alberta, Edmonton, AB T6G2R3, Canada.; ^4^ Deparment of Biochemistry, University of Alberta, Edmonton, AB T6G2R3, Canada.; ^5^ Department of Oncology, University of Alberta, Edmonton, AB T6G2R3, Canada.; ^6^ Nanostics Inc., Edmonton, Alberta T6N1H1, Canada.; ^7^ Centre for Prions and Protein Folding Diseases, University of Alberta, Edmonton, AB T6G2R3, Canada.; ^8^ Department of Medicine, University of Alberta, Edmonton, AB T6G2R3, Canada.

## Abstract

Gangliosides are glycosphingolipids with important roles in cell signaling and neuroprotection. While present on extracellular vesicles (EVs)—key mediators of intercellular communication—their role in EV biogenesis remains unclear. Here, we identify gangliosides as key modulators of EV biogenesis, with the specific composition of their glycan headgroup and the presence or absence of sialic acid and *N*-acetyl-d-galactosamine residues dictating whether they promote or inhibit EV biogenesis. GM1 and other complex gangliosides enhance EV secretion, while disruption of ganglioside synthesis impairs it. GM1 supplementation restores EV secretion in Huntington’s disease (HD) fibroblasts and cell models with ganglioside deficiency, including models of neurodegenerative diseases caused by a genetic block of ganglioside synthesis. Notably, GM1 enhances EV-mediated secretion of pathogenic misfolded proteins, including mutant huntingtin (mHTT), α-synuclein, and tau, reducing intracellular burden and providing mechanistic insight into the mHTT-lowering effects of GM1 in HD models. Our findings shed light on the neuroprotective roles of gangliosides and their therapeutic potential in misfolded protein disorders.

## Teaser

Gangliosides take on an unexpected job: Boosting extracellular vesicle release and shipping out harmful proteins in the process.

## INTRODUCTION

Extracellular vesicles (EVs) are membrane-bound particles ranging from 30 to 1000 nm in diameter, released by cells into the extracellular space (*1*). They originate either from intraluminal vesicles of multivesicular bodies (MVBs) when these fuse with the plasma membrane (exosomes) or from direct budding of the plasma membrane (ectosomes). EVs carry a large repertoire of macromolecules involved in their biogenesis and functions, enabling them to play critical roles in intercellular communication and the transfer of macromolecules and organelles between cells, under both physiological and pathological conditions (*1*–*4*). In the brain, EV-mediated molecular transfer and signaling influence development, synaptic plasticity, proteostasis, and microglia activity (*5*–*7*).

EVs also play a central role in misfolded protein diseases such as Alzheimer’s disease (AD), Parkinson’s disease (PD), and Huntington’s disease (HD). The release of EVs carrying pathogenic misfolded proteins helps alleviate proteotoxic stress in vulnerable cells and promotes the clearance of toxic proteins (*8*, *9*). However, EVs can also contribute to the intercellular transfer of misfolded proteins between neurons, promoting their prion-like spread throughout the brain (*10*–*13*). A central role of EVs in neurodegenerative diseases was further suggested by a large systems biology study that identified the EV pathway and protein network as among the most highly affected in multiple misfolded protein diseases (*14*). These findings raise critical questions about how misfolded protein diseases affect EV biogenesis and function and how these alterations may, in turn, influence the clearance and propagation of pathogenic proteins.

Neurodegenerative misfolded protein diseases are associated with substantial alterations in lipid metabolism (*15*), which contribute to both neurodegeneration and neuroinflammation (*16*–*18*). Many of the affected lipids play key roles in EV biogenesis (*19*), including ceramide, cholesterol, phosphatidylinositol, phosphatidylethanolamine, and phosphatidylserine (*20*–*22*). Notably, several proteins involved in EV biogenesis localize to membrane microdomains enriched with cholesterol, sphingolipids, and gangliosides (*23*–*26*). These microdomains serve as platforms where lipid composition influences protein binding, clustering, and activity, ultimately affecting EV biogenesis (*26*–*28*). Many of these lipids and proteins are incorporated and enriched into EVs themselves (*1*). Thus, lipid abnormalities in neurodegenerative diseases may disrupt EV biogenesis and functions, impairing EV-mediated cell-cell communication and proteostasis.

Gangliosides—sialic acid–containing glycosphingolipids highly abundant in the central nervous system (*29*, *30*)—are another key component of EV membranes (*31*). They localize to membrane microdomains rich in EV-associated lipids and proteins (*32*) and exhibit notable biophysical properties, including an amphiphilic nature, asymmetric distribution within membrane leaflets, and the ability to induce positive curvature in cell membranes—a feature shared by other EV-promoting lipids (*33*–*35*). These characteristics suggest a role for gangliosides in EV formation, yet this possibility remains largely unexplored. Addressing this knowledge gap is particularly relevant given that brain ganglioside levels are altered in several common neurodegenerative diseases (*29*, *36*–*40*). For example, ganglioside GM1 synthesis is decreased in PD and HD, while loss-of-function mutations in ganglioside biosynthetic enzymes result in the lack of complex gangliosides in a form of hereditary spastic paraplegia and other rare early-onset neurodegenerative disorders (*41*–*44*). Exogenous GM1 supplementation has been shown to alleviate symptoms (*40*, *45*–*48*) and reduce brain levels of mutant huntingtin (mHTT) (*45*) and α-synuclein (*49*) in models of HD and PD, respectively. However, whether altered ganglioside levels contribute to impaired EV secretion and how GM1 supplementation influences this process remains unknown.

Here, we describe a previously unidentified role for gangliosides, both endogenously synthesized and exogenously administered, in modulating EV secretion. We demonstrate that gangliosides influence EV release by neuronal and nonneuronal cells, either enhancing or reducing secretion depending on their specific glycan structure. Furthermore, we show that reduced cellular ganglioside levels impair the ability of cells to dispose of mHTT via the EV pathway, whereas GM1 administration enhances EV secretion, facilitating the export of misfolded proteins and decreasing intracellular mHTT burden. These findings provide insights into the neuroprotective effects of GM1 and uncover a previously unrecognized role for gangliosides in EV biogenesis, with extensive implications for the pathogenesis and treatment of neurodegenerative diseases.

## RESULTS

### Administration of ganglioside GM1 promotes the secretion of EVs from murine and human neuronal and nonneuronal cells

**Fig. 1.**
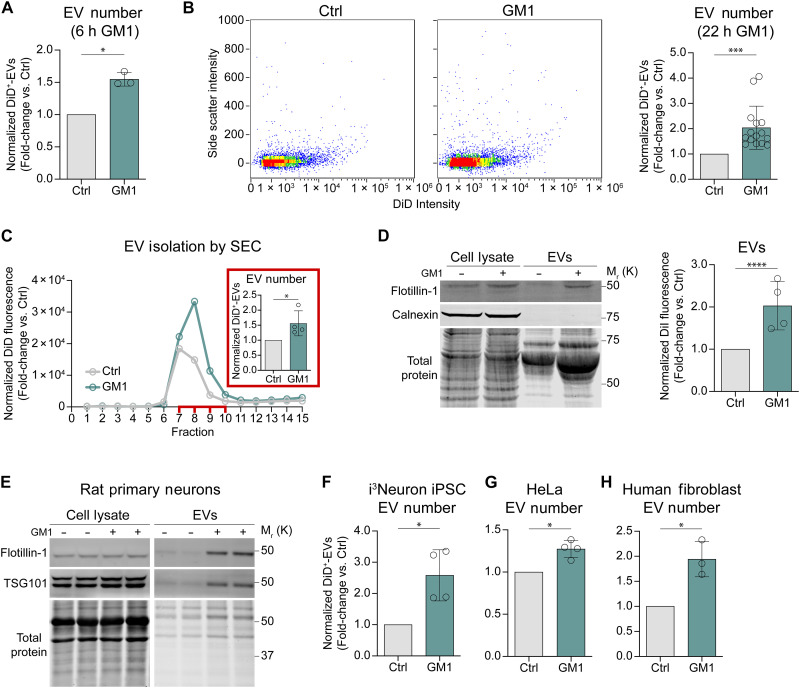
Administration of ganglioside GM1 promotes the secretion of EVs from murine and human cells of different origins. (**A**) N2a cells were treated with GM1 [50 μM, 6 hours (h)]. EVs were collected in serum-free medium containing N-2 for 16 hours and counted by IFC (*n* = 3 experiments). (**B**) Representative IFC dot plots and quantification of DiD^+^-EVs secreted by control or GM1-treated N2a cells. GM1 (50 μM) was present throughout a 22-hour EV collection (*n* = 15 experiments). (**C**) Representative size-exclusion chromatogram and quantification (inset) of EVs secreted by N2a cells treated with GM1 or vehicle for 6 hours. DiD fluorescence in each fraction was normalized to cellular protein. Peaks correspond to EV-rich fractions (7 to 10). Inset, DiD^+^-EVs in the EV-rich fractions measured by IFC and normalized to total cellular protein (*n* = 4 experiments). (**D**) N2a cells were treated with GM1 (50 μM, 18 hours), washed, and EVs collected for 24 hours were isolated by ultracentrifugation. (Left) Representative immunoblot for flotillin-1 (EVmarker) and calnexin (cell contamination control). (Right) DiI fluorescence in the EV pellet was measured by fluorometry and normalized to total cellular protein (*n* = 4 experiments). (**E**) DIV15 rat cortical neurons were treated with GM1 (50 μM, 72 hours). Equal fractions of ultracentrifugation-isolated EVs from control and GM1-treated neurons were immunoblotted (*n* = 2 independent cultures). (**F**) Human i^3^Neurons were treated with GM1 (50 μM) during a 22-hour EV collection (*n* = 4 experiments). (**G**) HeLa cells were incubated with GM1 (50 μM, 18 h), washed, and EVs collected for 24 h (*n* = 4 experiments). (**H**) Human fibroblasts from a healthy donor were treated with GM1 (50 μM, 6 hours), washed, and EVs collected for 16 hours (*n* = 3 experiments). [(B) and (E) to (H)] DiD^+^-EVs in CCM was measured by IFC and normalized to total cellular protein. Bars show means ± SD. Two-tailed paired *t* test. *P < 0.05, ***P < 0.001.

To assess the impact of gangliosides on EV secretion, we began by examining the effects of ganglioside GM1 on neuronal cells. GM1 administration results in ganglioside uptake and membrane enrichment in treated cells (*40*, *50*). To facilitate direct analysis of EVs secreted into the culture medium while minimizing sample handling and EV loss, we used a state-of-the-art imaging flow cytometry (IFC) platform optimized and validated for the analysis of fluorescent EVs (*51*, *52*) produced by cells prelabeled with a lipophilic dye [1,1′-dioctadecyl-3,3,3′,3′-tetramethylindodicarbocyanine (DiD) or 1,1′-dioctadecyl-3,3,3′,3′-tetramethylindocarbocyanine perchlorate (DiI), as indicated], which is incorporated in all cell membranes, including those of EVs (*53*). Apoptotic bodies and cell debris were removed from the conditioned medium by centrifugation at 2000*g* (cleared conditioned medium, CCM) before EV analysis (*53*). In our initial experiments, neuroblastoma N2a cells were incubated with 50 μM GM1 for 6 hours, followed by washing and EV collection in GM1-free collection medium for 16 hours. In these conditions, cells treated with GM1 secreted 50% more EVs compared to untreated controls (Fig. 1A and fig. S1). Notably, the increase in EV secretion was considerably higher when GM1 remained present throughout the 22-hour EV collection period (Fig. 1B). This effect is likely due to the continuous stimulatory action of GM1 on EV secretion, compared to conditions where the ganglioside was removed, and its cellular levels were allowed to return to baseline due to ganglioside catabolism and cell division. Similar results were observed when EVs were isolated from the CCM by size exclusion chromatography (SEC; Fig. 1C) or ultracentrifugation (Fig. 1D), two widely used methods (*54*, *55*). Western blot analysis of isolated EVs confirmed the expected enrichment of the EV marker Flotillin-1 (Fig. 1D).

Next, we sought to rule out the possibility that the increased number of EVs measured following GM1 treatment was due to reduced reuptake rather than enhanced secretion of EVs. To this end, we analyzed EV uptake in conditions that recapitulated those used for EV collection. DiD-labeled EVs isolated by SEC from control or GM1 treated N2a cells were incubated with either control or GM1-treated recipient (unlabeled) N2a cells in collection medium, and uptake was quantified by IFC. The GM1-rich EVs were internalized by GM1-rich cells to a similar extent as control EVs were by control cells (fig. S2). This indicates that the observed increase in EV number following GM1 treatment is most likely explained by increased EV secretion and cannot be attributed to reduced EV reuptake.

We then extended our investigations to determine whether the effects of GM1 on EV secretion are cell type– and species-dependent. GM1 treatment significantly increased EV secretion in primary rat neurons and human induced pluripotent stem cell (iPSC)–derived i^3^Neurons, as measured by EV isolation by ultracentrifugation and immunoblotting for the EV markers Flotilin-1 and TSG-101 (for rat neurons; Fig. 1E) and by IFC analysis of fluorescent EVs in the medium of DiD-labeled human i^3^Neurons (Fig. 1F). The stimulatory effects of GM1 on EV secretion also extended to nonneuronal cells, including HeLa cells (Fig. 1G) and primary human fibroblasts (Fig. 1H). Together, through complementary approaches for EV isolation and analysis, our data demonstrate that cell enrichment with GM1 enhances EV secretion in a wide range of cells, highlighting an important role for ganglioside GM1 in EV biogenesis.

### GM1 does not affect the size and tetraspanin profile of EVs but increases their GM1 content

**Fig. 2.**
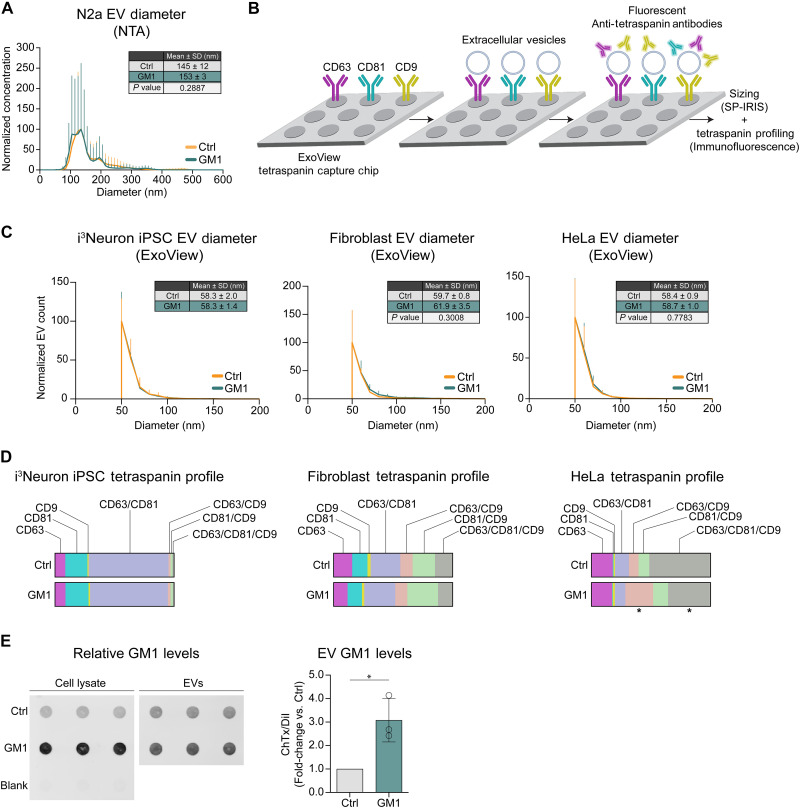
Cell treatment with GM1 preserves EV size and tetraspanin profile but increases EV GM1 content. (**A**) Particle size distribution profile of EVs secreted by N2a cells treated with GM1 compared to untreated cells, as measured by NTA. EV concentration was normalized to a common scale. The table reports the mean particle diameter ± SD and *P* values calculated by two-tailed paired *t* test (*n* = 3 experiments). (**B**) Schematic of the ExoView tetraspanin-capture chip to capture and profile EVs. EVs captured onto chips by anti-tetraspanin antibodies printed onto the chips are then labeled with fluorescent anti-tetraspanin antibodies and imaged for tetraspanin profiling and sizing by single-particle interferometric reflectance imaging (SP-IRIS). (**C**) Particle size distribution profile of EVs secreted by human i^3^Neurons (*n* = 2 experiments), human fibroblasts (*n* = 3 donor cell lines), and HeLa cells (*n* = 3 experiments) treated with GM1 compared to untreated cells, as detected by SP-IRIS. EV counts were normalized to a common scale. The table insets report the mean particle diameter ± SD and *P* values obtained by two-tailed paired *t* test. (**D**) Tetraspanin profile of EVs secreted by human i^3^Neurons (*n* = 2 experiments), human fibroblasts (*n* = 3 donor cell lines), and HeLa cells (*n* = 3 experiments) treated with GM1 compared to untreated cells. Colored bars show the proportion of EVs bearing the indicated tetraspanin combinations. *P < 0.05 by two-way analysis of variance (ANOVA) of probit-transformed data with Šídák’s post hoc test. (**E**) Representative dot blot of cholera toxin B binding to quantify GM1 in cell lysates and EVs from N2a cells treated with GM1 (50 μM, 6 hours), compared to untreated cells. GM1 was washed off, and EVs were collected for 16 hours and isolated by SEC. The graph shows the densitometric analysis of cholera toxin B binding to EV fractions, normalized to DiI EV fluorescence. Bars are means ± SD. *P < 0.05 by two-tailed paired *t* test (*n* = 3 experiments). (B) was created in BioRender Monyror, J. (2025) https://BioRender.com/r66v912.

To gain insights into the mechanism of action of GM1 and determine whether cell enrichment with GM1 affects a specific EV subpopulation—e.g., small versus large EVs with presumably different subcellular origin, or EV subpopulations characterized by specific tetraspanin profiles (*56*, *57*)—we analyzed EV size by nanoparticle tracking analysis (NTA) (*58*), dynamic light scattering (DLS) (*59*), and ExoView (*60*) and profiled EV tetraspanins using ExoView microchips (Fig. 2B) (*60*, *61*). NTA analysis of EVs in the CCM from GM1-treated and untreated N2a cells showed that GM1 treatment did not alter EV size distribution (Fig. 2A). The EVs detected were mostly small EVs with a diameter of around 150 nm, which is in the size range of both exosomes and ectosomes (*1*). We obtained similar results by DLS analysis after fractionating EVs by asymmetric flow field-flow fractionation (fig. S3A). GM1 treatment did not affect the size of EVs secreted by human iPSC-derived i^3^Neurons, human fibroblasts or HeLa cells (Fig. 2C), although, as expected, the average size of EVs measured in these cells by ExoView was smaller, likely because of EV dehydration during ExoView sample preparation (*62*).

Next, we analyzed the tetraspanin profile of EVs as this has been proposed to vary depending on the intracellular origin of EVs (*56*, *57*). While both human fibroblasts and i^3^Neurons produced EVs with quite different tetraspanin profiles, the proportion of EVs carrying the major tetraspanins CD63, CD81, and CD9 in different combinations was not significantly altered by GM1 treatment (Fig. 2D). This suggests that GM1 does not promote the secretion of specific EV subpopulations but rather enhances total EV secretion, maintaining the specific EV tetraspanin profile of each cell type. In HeLa cells, however, GM1 treatment increased the proportion of EVs bearing both CD63 and CD9 (from 8.1% in the medium of untreated cells to 23.4% of total EVs in the medium of GM1-treated cells), at the expense of CD63/CD9/CD81 triple-positive EVs (from 51.4 to 35.7%) (Fig. 2D). Thus, while GM1 promotes EV secretion in all cells, the specific subpopulation secreted might be cell type–dependent, suggesting that GM1 might affect fundamental shared mechanisms of EV biogenesis.

A major difference we observed between EVs secreted by GM1-treated cells and those from control cells was that the former were significantly enriched with GM1, mirroring a similar enrichment in their cells of origin (Fig. 2E).

### Inhibition of ganglioside synthesis impairs the ability of cells to secrete EVs

**Fig. 3.**
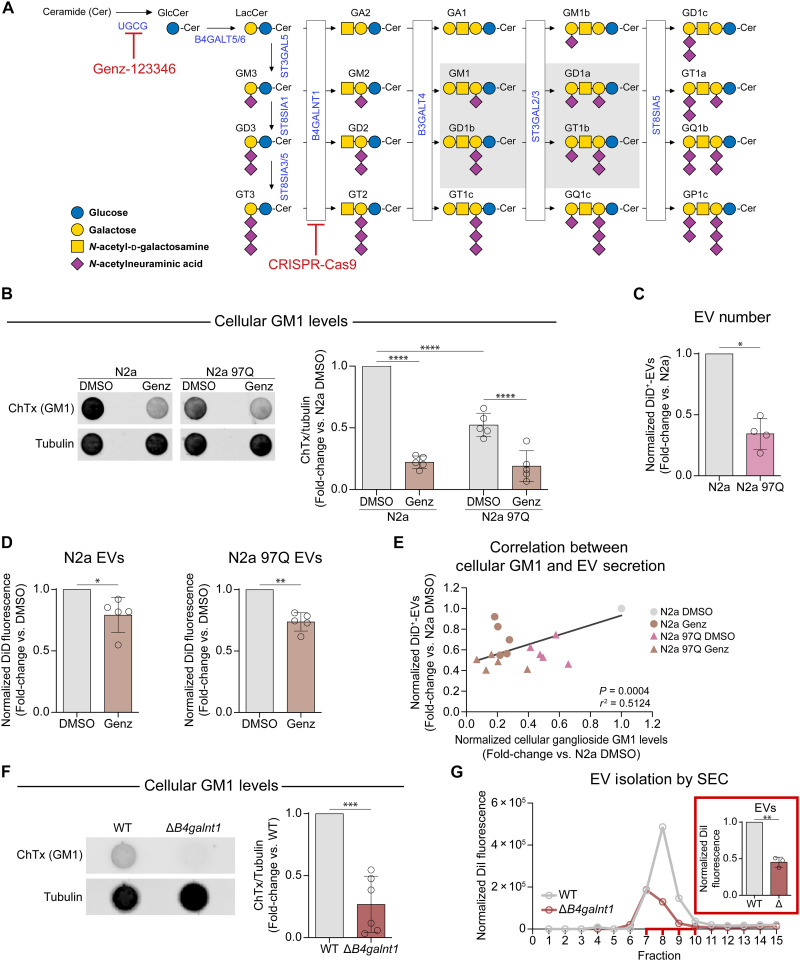
Lowered cellular ganglioside levels result in attenuated EV secretion. (**A**) The ganglioside biosynthetic pathway. Glycan moieties follow the Symbol Nomenclature for Glycans (SNFG). Enzymes in blue; the major brain gangliosides are boxed in gray. Genz-123346 inhibits GlcCer synthase; the gene product of *Ugcg*. *B4galnt1* was knocked-out using CRISPR-Cas9. [Adapted from Sipione *et al.* (*29*)]. (**B**) Representative dot-blot and densitometric analysis of cholera toxin binding to lysates from N2a and N2a 97Q cells lysates after 48 hours of treatment with 1 μM Genz-123346, to assess GM1 levels. Tubulin was the loading control (*n* = 5 experiments). (**C**) Fold-change in DiD^+^-EV secretion by mHTT-expressing N2a 97Q versus N2a cells, measured by IFC and normalized to cellular protein (*n* = 4 experiments). (**D**) DiD^+^- EV fluorescence in the CCM of N2a (left) and N2a 97Q (right) cells after 48 hours of 1 μM Genz-123346 treatment, normalized to cellular protein (*n* = 5 experiments). (**E**) Pearson correlation between cellular GM1 levels (quantified by cholera toxin binding and normalized to tubulin) and EV secretion (measured by IFC and DiD^+^-EV number normalized to total cellular DiD fluorescence) in N2a and N2a 97Q cells treated with DMSO (vehicle) or Genz-123346 for 48 hours (*n* = 5 experiments). (**F**) Dot-blot and densitometric analysis of cholera toxin binding to assess GM1 in N2a and N2a Δ*B4galnt1* cell lysates after 22 to 24 hours in serum-free medium containing N-2. Tubulin was used as the loading control (*n* = 6 experiments). (**G**) Representative size-exclusion chromatogram of EVs secreted by N2a and N2a Δ*B4galnt1* cells. DiI fluorescence in each fraction was normalized to cellular DiI. Peaks represent EV-rich fractions (7 to 10). Inset, DiI^+^-EVs in the EV-rich fractions measured by IFC and normalized to cellular DiI (*n* = 3 experiments). Bars show means ± SD. *P < 0.05, **P < 0.01, ***P < 0.001, ****P < 0.0001 by two-way ANOVA with Tukey’s post hoc test (B) or two-tailed paired *t* test [(C), (D), (F), and (G)].

To investigate the role of endogenously synthesized gangliosides and shed light on the potential implications of decreased ganglioside levels in neurodegenerative diseases, we used three different models of cellular ganglioside reduction: (i) N2a cells treated with Genz-123346, a pharmacological inhibitor of glucosylceramide (GlcCer) synthase (*63*), the first enzyme committed to the synthesis of gangliosides (Fig. 3A). This treatment only partially depletes gangliosides and thus mimics the partial reduction of gangliosides that is observed in HD (*36*, *64*) and other pathophysiological conditions (*65*–*67*); (ii) N2a cells overexpressing an N-terminal (exon 1) fragment of mHTT (N2a 97Q cells), an HD model that recapitulates the decrease in GM1 cellular levels described in other HD models and patients’ cells (*39*, *40*); (iii) knockout (KO) of *B4galnt1* in N2a cells by CRISPR-Cas9 (*68*), to block the synthesis of complex gangliosides (Fig. 3A) and model a defect of ganglioside biosynthesis in humans (*41*, *42*). As expected, we observed significantly lower levels of GM1 in N2a 97Q cells compared to wild-type (WT) N2a cells, given that expression of mHTT decreases ganglioside levels across multiple animal and cell models (*36*, *40*). GM1 levels were further lowered in cells of both genotypes by treatment with 1 μM Genz-123346 (Fig. 3B). Concomitantly, N2a 97Q cells secreted fewer EVs than WT N2a cells (Fig. 3C). A further impairment in the secretion of EVs by cells of both genotypes was observed upon inhibition of ganglioside synthesis with Genz-123346 (Fig. 3D and fig. S4, A and B), revealing the existence of a positive correlation between EV secretion and intracellular levels of GM1 (Fig. 3E). In line with these conclusions, blocking GM1 synthesis by KO of *B4galnt1* (Fig. 3F) resulted in a >50% decrease in EV secretion compared to control cells (Fig. 3G and fig. S4, C and D).

### Administration of exogenous gangliosides restores EV secretion defects in *B4galnt1* KO cells and in HD cells

**Fig. 4.**
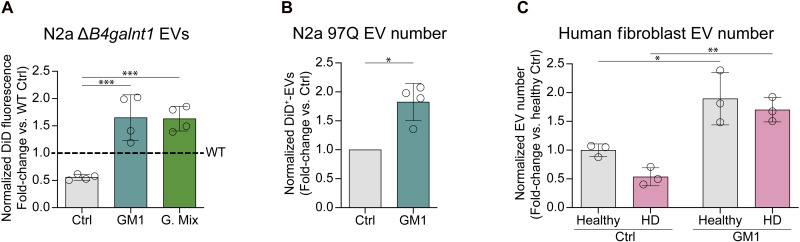
GM1 administration rescues EV secretion in cells with impaired ganglioside synthesis. (**A**) Total EV fluorescence in the CCM of N2a Δ*B4galnt1* cells incubated with 50 μM GM1 or a 50 μM equimolar mixture of the major brain gangliosides (G. Mix: GM1, GD1a, GD1b, and GT1b) for 22 hours (*n* = 4 experiments). (**B**) Number of DiD^+^-EVs secreted by N2a 97Q cells treated with 50 μM GM1 for 6 hours compared to untreated cells (*n* = 4 experiments). (**C**) Number of EVs secreted by primary human fibroblasts from three healthy controls and three age-matched patients with HD incubated 50 μM GM1 for 22 hours, compared to untreated controls. EVs were captured and analyzed using the Exoview platform. Circles represent the data from different donors. EV fluorescence and numbers were normalized to total cellular protein content. Bars indicate means ± SD. *P < 0.05, **P < 0.01, ***P < 0.001 by one-way ANOVA with Tukey’s post hoc test (A), two-tailed paired *t* test (B), or two-way ANOVA with Tukey’s post hoc test (C).

Reduced EV secretion in *B4galnt1* KO cells could be restored by administration of an equimolar mix (17.5 μM each) of the four major gangliosides (GM1, GD1a, GD1b, and GT1b) that are downstream of the synthetic block in *B4galnt1* KO cells, or even 50 μM GM1 alone (Fig. 4A). Akin to the rescue of EV secretion in *B4galnt1* KO cells, supplementation of N2a 97Q cells with GM1 resulted in a doubling of EVs secreted in the culture medium (Fig. 4B). To further investigate the defect of EV secretion in HD models and whether it can be rescued by ganglioside administration, we analyzed EV secretion in primary human fibroblasts from three healthy controls and three age-matched HD carriers. All three HD lines—previously shown to have lower GM1 levels than control fibroblasts (*40*)—secreted less EVs than healthy fibroblasts (unpaired two-tailed *t* test, *P* = 0.0133), recapitulating the correlation between lowered GM1 levels and impaired EV secretion described for N2a 97Q cells. This reduction was driven by decreased secretion of CD81^+^ and CD63^+^/CD9^+^ double-positive EVs (fig. S5). Administration of GM1 increased EV secretion from all lines and restored EV secretion in HD cells to control fibroblast levels (Fig. 4C). GM1 treatment did not alter the size of EVs secreted by N2a 97Q or HD fibroblasts, in line with our results in WT cells (fig. S3, B to D). Together, our findings suggest that cell gangliosides are key modulators of EV secretion, with decreased levels or lack of gangliosides resulting in impaired EV secretion in disease models, and supraphysiological levels of GM1 (as obtained upon GM1 administration) promoting EV secretion and rescuing EV biogenesis defects.

### Secretion of misfolded proteins within EVs is affected by the cellular ganglioside content

**Fig. 5.**
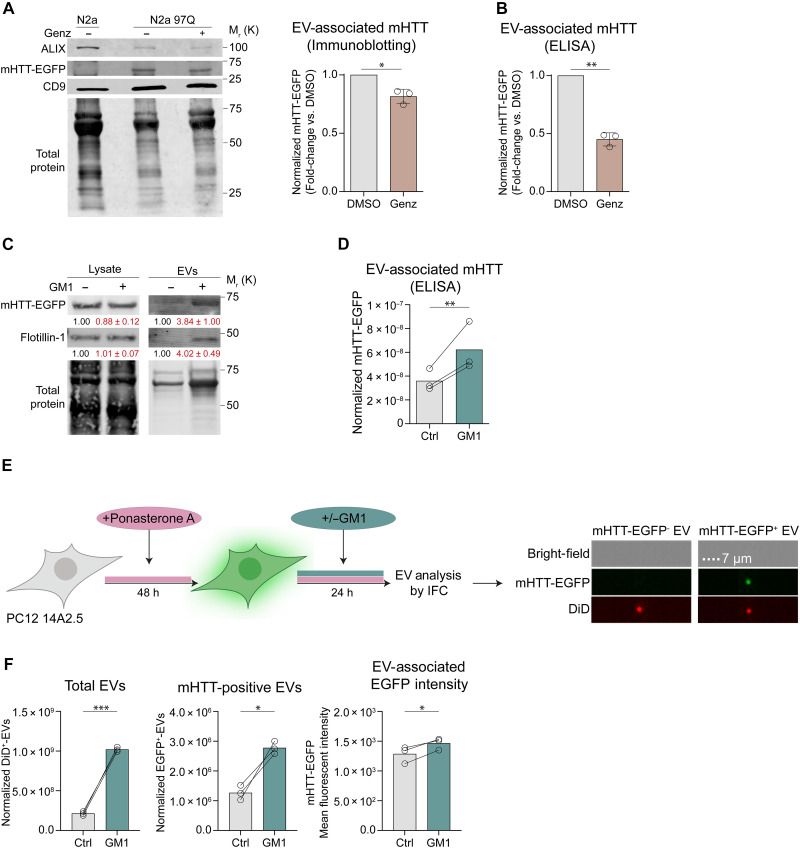
Secretion of mHTT within EVs is modulated by the cellular ganglioside content. (**A**) (Left) Representative immunoblots of EVs isolated by SEC from the conditioned medium of N2a 97Q cells treated or not with 1 μM Genz-123346 for 48 hours. EVs were collected in the last 24 hours of incubation. ALIX and CD9 are EV markers. EV-associated mHTT-EGFP was quantified by densitometry (graph on the right; *n* = 3 experiments). (**B**) mHTT-EGFP in EVs was quantified by ELISA and normalized to total cellular protein content (*n* = 3 experiments). (**C**) Representative immunoblot of mHTT-EGFP in N2a 97Q cell lysates and EVs isolated by ultracentrifugation after cell treatment with GM1 (50 μM, 18 hours), followed by EV collection for 24 hours. Flotillin-1 is an EV marker. Densitometric values (means ± SD) are shown below each band. Data were normalized over total cellular protein for EVs (*n* = 2 experiments) or total protein stain for lysates (*n* = 3 experiments). (**D**) ELISA quantification of mHTT-EGFP in SEC-isolated EVs from N2a 97Q treated with GM1 (50 μM, 22 hours). Data were normalized to total cellular protein (*n* = 3 experiments). (**E**) (Left) PC12 14A2.5 cells were induced with ponasterone A for 72 hours to express mHTT-EGFP and treated with GM1 (50 μM) for the last 24 hours. EVs in the CCM were analyzed by IFC. (Right) Representative IFC images of mHTT-EGFP^−^- and mHTT-EGFP^+^-EVs. (**F**) Quantitation of total EVs (DiD^+^, left), mHTT^+^-EVs (DiD^+^/EGFP^+^, middle), and their mean EGFP intensity (right) by IFC (*n* = 3 experiments). EV counts were normalized to total cellular protein. Bars show means ± SD. *P < 0.05, **P < 0.01, ***P < 0.001 by two-tailed paired *t* test [(A), (B), and (F)] or ratio paired *t* test (D). (E) was created in BioRender. Monyror, J. (2025) https://BioRender.com/nuqysro.

Misfolded proteins, including pathogenic proteins such as mHTT, can be secreted as EV cargo to alleviate cell proteotoxic stress (*8*, *69*). Therefore, defects in EV secretion could affect the ability of cells to dispose of misfolded proteins via EVs. N2a 97Q treated with 1 μM Genz-123346 cleared less mHTT via EVs compared to untreated cells, as evidenced by the amount of mHTT in EV fractions isolated by SEC, measured by immunoblotting (Fig. 5A) and enzyme-linked immunosorbent assay (ELISA) (Fig. 5B). In contrast, cell treatment with GM1 promoted the release of mHTT with EVs (Fig. 5, C and D). The mHTT detected in EV fractions was located within the EV particles, similar to ALIX, a classic intralumenal EV marker. This was determined in a proteinase K protection assay that showed that mHTT (and ALIX) could only be degraded by the protease after EV membrane permeabilization with a detergent, to allow the enzyme to reach intravesicular cargo (fig. S6). This suggests that mHTT is in the EV lumen, not on the EV surface.

**Fig. 6.**
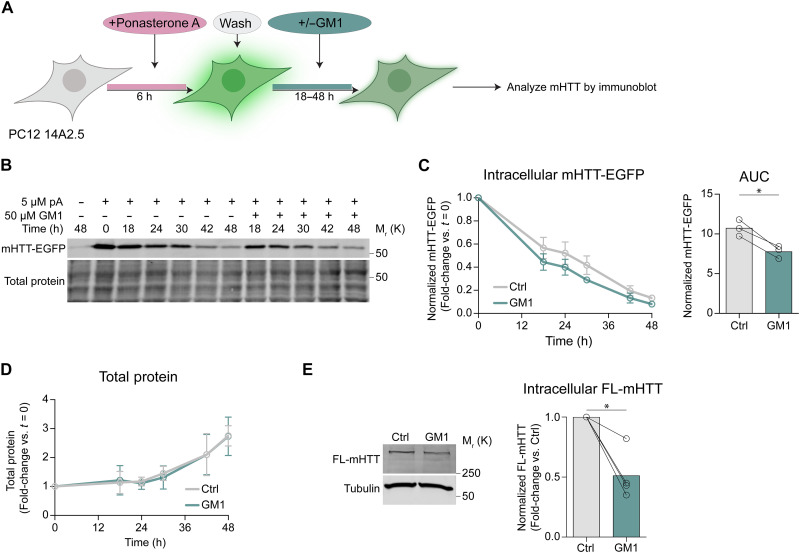
Intracellular mHTT levels are reduced following GM1 treatment. (**A**) PC12 14A2.5 cells were induced to express mHTT-EGFP with ponasterone A for 6 hours. Ponasterone A was then washed off to allow to follow mHTT clearance over time in untreated cells and cells incubated with 50 μM GM1 for up to 48 hours. (**B**) Representative immunoblot of the time course of mHTT-EGFP clearance after withdrawal of ponasterone A. (**C**) Densitometric analysis of mHTT-EGFP levels normalized to total protein stain and quantitation of cumulative mHTT-EGFP burden (area under the curve, AUC) (*n* = 3 experiments). (**D**) Total protein quantification in lysates of Ctrl and GM1-treated PC12 14A2.5 cells over 48 hours (*n* = 3 experiments). (**E**) Representative immunoblot and densitometric analysis of intracellular full-length (FL) mHTT in STHDh Q111/111 cells following treatment with 50 μM GM1 for 48 hours compared to untreated cells (*n* = 4 experiments). Bars show mean values ± SD. *P < 0.05 by two-tailed paired *t* test. (A) was in BioRender. Monyror, J. (2025) https://BioRender.com/76b8sqh.

To determine whether the overall increase in mHTT levels in EV fractions following GM1 treatment was due to enhanced mHTT loading into individual particles or a higher number of EVs carrying the protein, we performed IFC analysis of EVs secreted by PC12 cells that express mHTT–enhanced green fluorescent protein (EGFP) upon induction with ponasterone A (*70*). These cells express higher levels of mHTT-EGFP compared to N2a 97Q, allowing for the specific detection of EGFP^+^-EVs (carrying mHTT-EGFP) and for the relative quantification of EGFP fluorescence within each vesicle by IFC (Fig. 5E). GM1 treatment not only increased the number of EGFP^+^-EVs released in the medium but also increased their mean EGFP fluorescence intensity (Fig. 5F), suggesting that GM1 enhances both the loading of mHTT into individual EVs and the number of EVs carrying mHTT. Furthermore, this increase in the release of mHTT via EVs correlated with a lower intracellular mHTT burden, as shown by the accelerated clearance of intracellular mHTT over time (Fig. 6, A to C). Total protein levels were not affected by GM1 (Fig. 5D), suggesting that the observed decrease in mHTT levels was not due to cell death or GM1 effects on general proteostasis. Decreased intracellular mHTT levels were also observed upon GM1 incubation with STHDh^111/111^ cells (Fig. 6E), a striatal HD knock-in model that expresses full-length mHTT from the endogenous *Htt* locus (*71*).

**Fig. 7.**
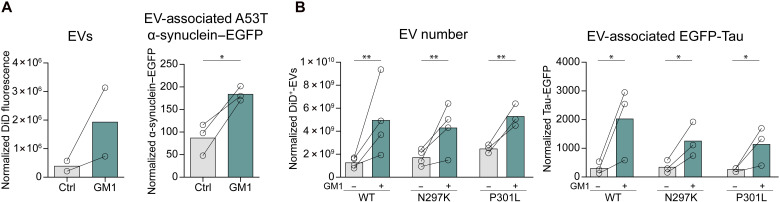
GM1 promotes cellular secretion of α-synuclein and tau through EVs. (**A**) N2a cells expressing A53T α-synuclein–EGFP were incubated with 50 μM GM1 for 22 hours in phenol red– serum-free medium supplemented with N-2. EVs were isolated by SEC and quantified by fluorometry (left). EV-associated A53T α-synuclein–EGFP was measured by ELISA (right). Data were normalized to total cellular protein content (*n* = 3 independent experiments). (**B**) WT GFP-Tau, N279K GFP-Tau, or P301L GFP-Tau expression was induced in HEK293T cells with doxycycline (10 ng/ml) for 72 hours. Cells were then incubated with 50 μM GM1 for 22 hours; EVs were isolated by SEC and quantified by IFC (left). EV-associated GFP-Tau was measured by ELISA (right). Data were normalized to total cellular protein content (*n* = 3 experiments). Bars show means. *P < 0.05, **P < 0.01 by paired *t* test (A) or two-way ANOVA with Tukey’s post hoc test (B).

The ability of GM1 to promote protein clearance via EVs was not limited to mHTT but extended to other disease models and pathogenic proteins, including N2a cells expressing A53T α-synuclein and human embryonic kidney (HEK) cells expressing either WT Tau or its disease-associated variants N297K and P301L (Fig. 7). Together, our data suggest that through modulation of EV release, gangliosides can affect the disposal of pathogenic proteins via EVs and overall intracellular misfolded protein burden.

### Sialic acid, *N*-acetyl-d-galactosamine, and ceramide are key molecular determinants of the modulatory effects of gangliosides on EV secretion

**Fig. 8.**
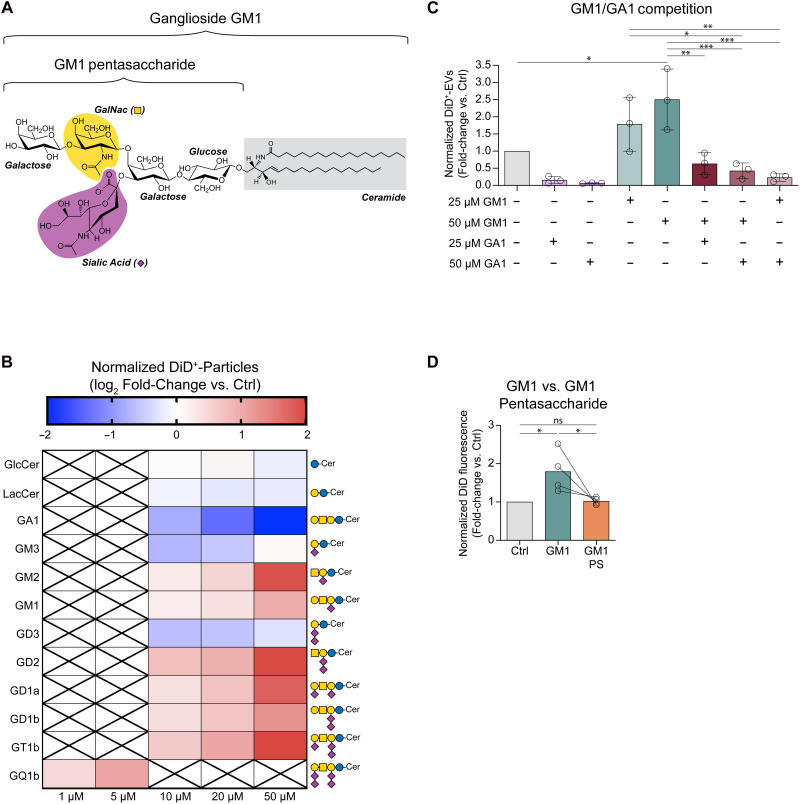
The effects of gangliosides on EV secretion depend on their ceramide tail and the presence of sialic acid and *N*-acetyl-d-galactosamine in their headgroup. (**A**) Structure of ganglioside GM1 as a prototypical ganglioside. *N*-acetyneuraminic acid (sialic acid), *N*-acetyl-d-galactosamine (GalNac), and the ceramide tail are highlighted in purple, yellow and gray, respectively. The SNFG symbols for sialic acid and GalNac are shown in brackets beside the sugar residue name. (**B**) Heatmap of the log_2_ fold-change of the number of EVs secreted from N2a cells treated with different gangliosides and glycosphingolipids at the indicated concentrations for 22 hours compared to their respective controls. Blue shades indicate decreased EV secretion, and red shades indicate increased EV secretion. The SNFG structures of each tested ganglioside and glycosphingolipid are shown on the right of the heatmap. The number of EVs in the CCM was measured by IFC and normalized to total cellular protein content (*n* = 3 to 5 experiments). GlcCer and LacCer did not significantly affect EV secretion (*P* > 0.05 by Friedman’s test). All ganglioside treatments significantly affected EV secretion (*P* < 0.05 by Friedman’s test). (**C**) N2a cells were treated with ganglioside GM1 or GA1 individually or together at the indicated concentrations for 22 hours in phenol red- serum–free medium supplemented with N-2. The number of EVs in the CCM was measured by IFC and normalized to total cellular protein content (*n* = 3 experiments). (**D**) Fluorescence of EVs secreted by N2a cells treated with 50 μM GM1 or GM1 pentasaccharide (GM1 PS) for 22 hours. EV fluorescence was normalized to total cellular protein (*n* = 5 experiments). Heatmap cells are means. Bars represent means ± SD. *P < 0.05, **P < 0.01, ***P < 0.001 by Friedman’s test (B), repeated measures one-way ANOVA with Tukey’s post hoc test (C), or one-way ANOVA with Tukey’s post hoc (D).

Our studies on EV secretion have so far focused on the effects of a specific ganglioside, GM1, or pharmacological and genetic interventions that broadly reduce the levels of most or all gangliosides, including GM1. While all gangliosides share a common structure—a ceramide lipid tail attached to a variable glycan headgroup (Fig. 8A)—the composition of the glycan headgroup not only defines individual gangliosides but also dictates their distinct behaviors, functions, and interactions (*72*), which can be remarkably different among gangliosides. Therefore, we aimed to investigate whether the ability of GM1 to promote EV secretion is exclusive or a general feature of all gangliosides, regardless of glycan headgroup composition.

To address this question, we tested the effects of all major gangliosides and two ganglioside precursors, GlcCer and lactosylceramide (LacCer), on EV secretion. All complex gangliosides stimulated EV secretion in N2a cells, with those carrying more sialic acid residues, including GD2, GD1a, GD1b, and GT1b, being more effective, and GQ1b being more potent than GM1 (Fig. 8B). In contrast, the simpler gangliosides GM3 and GD3 inhibited EV secretion, while the ganglioside precursors GlcCer and LacCer did not significantly affect it. Strikingly, ganglioside GA1 (asialo-GM1), which shares the glycan structure of GM1 but lacks the sialic acid residue, was a very effective inhibitor of EV secretion (Fig. 8B). Furthermore, GA1 abrogated the EV-promoting effects of GM1, reducing EV secretion to very low levels in both GM1-treated and untreated N2a cells (Fig. 8C). These findings demonstrate that the ability to promote EV secretion is not a universal property of all gangliosides and depends on specific ganglioside glycan structures. In particular, the distinct behaviors of simple (GM3 and GD3, which inhibit EV secretion) versus complex gangliosides (which promote EV secretion) suggest that the presence of the *N*-acetyl-d-galactosamine (GalNac) residue in complex gangliosides may be a critical determinant of the EV-promoting effects of gangliosides. The presence of at least one sialic acid residue also appears essential, as its absence reverses the ganglioside effects on EV secretion.

Gangliosides localize to membrane microdomains, and their functions often require or are mediated by their ceramide tail (*33*). To determine whether this is also true for their EV-promoting activity, we treated N2a cells with either GM1 or the GM1 pentasaccharide, which shares the same glycan structure of GM1 but lacks its ceramide moiety. The pentasaccharide was unable to recapitulate the effects of GM1 on EV secretion (Fig. 8D), suggesting that membrane anchoring via the ceramide tail is necessary for gangliosides to modulate EV secretion.

## DISCUSSION

Gangliosides are multifunctional molecules with numerous roles in both health and disease (*29*, *30*). Here, we have identified a previously unrecognized function of GM1 and other gangliosides in modulating EV secretion. The modulatory effects of gangliosides on EV biogenesis may have important implications in neurodegenerative diseases where levels of gangliosides are altered and may help explain some of the previously described therapeutic effects of GM1 in models of misfolded protein diseases (*45*, *47*, *49*).

Using several complementary approaches, we have shown that GM1 administration to neuroblastoma N2a cells stimulates EV secretion. This effect is further enhanced with continuous administration of the ganglioside. To minimize sample loss and avoid biases introduced by EV isolation methods that rely on specific markers—the expression of which may vary across cell types and treatments—we initially used direct IFC analysis of in-cell fluorescently labeled EVs (*53*). We then confirmed our findings by quantifying EVs isolated by ultracentrifugation or SEC, the two most widely used EV isolation methods (*55*, *73*). The GM1-induced increase in the number of EVs secreted was mirrored by a rise in total protein and EV markers in EV preparations from both N2a cells and primary rat neurons, confirming that we measured bona fide EVs.

GM1 significantly increases EV secretion not only in immortalized and primary neuronal cells from mice and rats but also in human cell lines (HeLa and HEK293 cells), primary human skin fibroblasts, and human iPSC-derived neurons. The ability of GM1 to increase EV secretion across species, cell types, or tissue origins suggests a fundamental role for gangliosides in EV production. In contrast, disruption of endogenous ganglioside biosynthesis through pharmacological (Genz-123346) or genetic (CRISPR-Cas9 KO of *B4galnt1*) approaches significantly decreases EV secretion, with cellular GM1 levels positively correlating with the amount of secreted EVs. This finding suggests that it is not only the supraphysiological cellular ganglioside levels achieved by pharmacological administration of GM1 that promotes EV secretion, but rather endogenous gangliosides may play an integral role in constitutive EV secretion. Supporting these conclusions, KO of *B4galnt1* (encoding GM2/GD2 synthase*)* in N2a cells significantly decreased EV secretion, whereas in another study, overexpression of *B3GALT4* (encoding GM1 synthase) in a breast cancer cell line increased levels of the EV markers TSG101 and CD63 in EV fractions, suggesting enhanced EV release (*74*).

Although our study did not directly examine the effects of gangliosides on EV secretion in microglia, astrocytes, or other brain cells, it is likely that gangliosides exert similar effects in these cells as in neurons, given the broad activity of GM1 across diverse cell types and species. Whether GM1 treatment preferentially enhances the production of exosomes or ectosomes remains to be determined. We found that GM1 did not specifically increase secretion of small or large EVs as EV size distribution remained unchanged across the various cell types analyzed. Some studies have attempted to differentiate EVs (exosome versus ectosome) on the basis of their tetraspanin profiles. While CD63 has been proposed as a specific marker for exosomes (*56*), recent findings in HeLa cells suggest a more complex relationship between EV origin and tetraspanin profile, with CD63, CD9, and CD81 all transiently localizing to both MVBs and the plasma membrane following translation, ruling out the presence of any single tetraspanin on a vesicle as a marker of its origin (*75*). Regardless of the baseline tetraspanin profile of EVs secreted by different cell types, which was different from one cell type to another, GM1 treatment proportionately increased all EV subtypes, suggesting a general mechanism of action rather than selective modulation of a specific EV subpopulation.

To investigate the structural requirements underlying the EV-promoting activity of GM1, we examined how different ganglioside features influence EV secretion. Both the specific composition of the glycan headgroup and the presence of the ceramide lipid tail proved essential. The GM1 pentasaccharide alone failed to enhance EV secretion, implying that ganglioside incorporation into membranes through their ceramide tail is required. The integration of gangliosides into cell membranes dictates their intracellular traffic and subcellular localization and affects membrane properties and curvature (*33*, *76*). Of note, GM1 and other gangliosides induce positive membrane curvature (*33*, *34*), which might promote EV biogenesis both at the plasma membrane and in MVB through the formation of intraluminal vesicles, similar to the well-established effects of ceramide on exosome biogenesis (*77*). However, membrane curvature alone is unlikely to fully explain the effects of gangliosides on EV secretion. Catabolism of gangliosides to EV-promoting lipids like ceramide and sphingosine (*78*, *79*) is also an unlikely mechanism. While most gangliosides induce positive membrane curvature, although to different extents (*33*), and all gangliosides are catabolized to ceramide and sphingosine, our data clearly show that the specific glycan structures modulate both the magnitude and direction of their effect on EV secretion. This strongly suggests that gangliosides influence EV secretion through the molecular interactions of their glycan headgroups rather than via their shared sphingolipid catabolites.

Notably, gangliosides with higher sialic acid content—typically associated with greater membrane curvature and stronger segregation properties in lipid bilayers (*33*)—were more effective than GM1 in promoting EV secretion. In contrast, GM3 and GD3 inhibited EV release. In addition, GA1, a ganglioside structurally similar to GM1 but lacking sialic acid, strongly inhibited EV secretion in a dose-dependent manner. Furthermore, coadministration of GA1 and GM1 abolished the EV-promoting effect of GM1, resembling a dominant-negative interaction. Since GA1 induces positive membrane curvature to a degree comparable to GM1 (*80*) yet suppresses EV secretion, our findings further support the conclusion that the effects of gangliosides on EV biogenesis are mainly driven by specific sugar residues rather than membrane curvature alone. The inhibitory effects of GM3 and GD3, which lack GalNAc in their headgroup, suggest that both sialic acid and GalNAc residues are required for the positive effects of gangliosides on EV secretion. We propose that gangliosides may engage protein partners involved in EV biogenesis via these residues and that partial engagement with only one of these sugars might hamper EV biogenesis. Alternatively, intramolecular interactions between the sialic acid and the GalNAc residue, which confer a more rigid conformation to the headgroup of complex gangliosides (*33*), may be necessary for the interaction with partners in EV biogenesis and their functional effects. In support of these hypotheses, the ganglioside precursors GlcCer and LacCer, which lack both sialic acid and GalNAc residues, had no significant effect on EV secretion in our model.

Many proteins directly implicated in EV biogenesis, including ESCRT components, flotillin-1, syndecan-1, and tetraspanins, localize to membrane microdomains enriched with gangliosides (*23*–*27*, *81*). A recent study using a clickable photoaffinity GM1 probe identified CD9 and other proteins involved in EV biogenesis as part of the human GM1 interactome (*82*). Therefore, interactions between gangliosides and EV biogenesis proteins may underlie their modulatory effects on EV secretion, by stabilizing or sequestering factors in lipid rafts, altering their conformation and activity, or modulating their interactions with partner proteins (*83*–*87*). Thus, gangliosides may fine-tune vesicle formation and release by shaping the molecular environment where EV biogenesis occurs.

Our finding raises important questions about how EVs are affected by aging, when brain levels of complex gangliosides decline (*88*), and in pathologies where ganglioside metabolism is disrupted. The N2a *B4galnt1* KO cells used in our study model the ganglioside biosynthetic defect resulting from mutations in the human B4GALNT1 gene, which causes a form of hereditary spastic paraplegia (*41*). The impaired EV secretion observed in these cells may reflect a similar defect in patient-derived cells and contribute to disease pathogenesis, given the role of EVs in processes that are key to normal brain function, including development, synaptic plasticity, proteostasis, and microglia activity (*5*–*7*).

In HD, mHTT expression reduces cellular and brain levels of GM1 and other major gangliosides (*36*, *40*), a finding replicated in the HD cell models used in this study. We show that HD cells (N2a 97Q and primary fibroblasts) secrete fewer EVs than controls, a phenotype reversed by GM1 administration. Together with prior work (*89*), our data experimentally confirm impaired EV secretion in HD and support previous computational predictions (*14*). However, they are in contrast with another study reporting increased EV secretion from HD fibroblasts and derived neural stem cells (*90*). The discrepancy may stem from differences in EV isolation and analytical methods used, as Beatriz *et al.* (*90*) focused their analysis on small EVs isolated by ultracentrifugation, a method that may result in EV losses (*53*, *91*) and might bias results, while our approach minimizes sample loss and captures a broader EV population. Although multiple mechanisms might be responsible for the changes in EV secretion observed in HD cells (*89*), our data suggest that the reduction in ganglioside levels is key since the phenotype is rescued by GM1 administration.

EVs carrying misfolded proteins play important roles in brain proteostasis (*8*, *69*, *92*), reducing the intracellular accumulation of pathogenic proteins and mitigating their toxicity (*8*, *9*, *69*). Thus, impaired EV production in HD cells could have a negative impact not only on brain processes modulated by EVs but also on the cells’ ability to export and clear mHTT through this pathway. Our studies support this hypothesis by showing that pharmacological inhibition of ganglioside synthesis with Genz-123346 decreases the amount of mHTT secreted via EVs. In contrast, GM1 treatment increases the secretion of EV-associated mHTT in a neuronal model and human HD fibroblasts. A proteinase K protection assay allowed us to determine that mHTT is loaded into EVs as a luminal cargo rather than associated to their surface. In an inducible PC12 cell model, GM1 treatment increases both the number of mHTT-containing EVs released in the medium and the amount of mHTT loaded into each particle while concomitantly decreasing the intracellular mHTT burden. These results highlight the therapeutic potential of gangliosides and align with and contribute to explain the mHTT-lowering effects of GM1 in vivo in HD mouse model, which result in neuroprotection and restoration of normal motor and nonmotor behavior (*45*, *46*). Given the greater effectiveness of other gangliosides (GD2, GD1a, GD1b, and GT1b) compared to GM1 in promoting EV secretion, the question arises whether they might confer even stronger neuroprotective effects. However, GM1’s overall neuroprotective effects might include a range of mechanisms beyond EV secretion (*93*), which might not be shared by other gangliosides.

Beyond HD, GM1 promotes EV-mediated secretion of A53T α-synuclein and WT and mutant tau—pathogenic proteins implicated in familial PD, AD, and tauopathies, respectively—suggesting broader therapeutic potential. Further studies are needed to elucidate the mechanism by which gangliosides influence misfolded protein packaging into EVs. One possibility is that their accumulation at the membrane of MVBs might facilitate the recruitment of chaperones and adaptor proteins responsible for cargo loading. For example, the cysteine-string protein (CSPα or DnaJC5) was shown to play a role in mHTT loading into 180 to 240 nm and larger vesicles (*94*), but whether it is involved in the effects of GM1 described in our studies remains to be determined.

A potential concern regarding the use of gangliosides to enhance misfolded protein secretion via EVs is whether this could promote the prion-like spreading of pathogenic proteins throughout the brain (*10*, *12*). However, this appears unlikely, given strong experimental evidence from in vivo models demonstrating that GM1 administration slows or even halts neurodegeneration, lowering brain levels of mHTT and α-synuclein (*45*, *46*, *49*). We speculate that GM1 not only increases EV production but also modifies their fate. In addition to increasing cellular ganglioside levels, GM1 treatment increases its own levels in secreted EVs. This may have considerable consequences for EV fate and function, as gangliosides reside in the outer leaflet of EV membranes and may contribute to recognition, interaction, and uptake by target cells. Gangliosides on EVs have been implicated in EV internalization through interactions with integrins and sialic acid–binding siglec receptors on recipient cells in various tissues and cell types (*68*, *95*). In the brain, EV enrichment with GM1 could facilitate uptake and enhance degradation of misfolded protein cargo by microglia, preventing prion-like propagation and explaining why GM1 administration in HD mice reduces brain mHTT levels and mitigates neurodegeneration. Beyond acting as a ligand for EV uptake, gangliosides on the EV surface may also have signaling roles. For example, EV-associated GD3 mediates T cell arrest in the ovarian tumor microenvironment (*89*). Similarly, GM1-rich EVs secreted by brain cells could influence microglial activity and dampen proinflammatory responses, consistent with our findings using ganglioside-containing liposomes and micelles to treat microglia (*96*, *97*). Given that depletion of cellular gangliosides—either pharmacologically (Genz-123346) or genetically (CRISPR-Cas9 KO of *B4galnt1*)—reduced ganglioside content in secreted EVs, modulating cellular ganglioside levels could represent a method to alter EV-mediated signaling and cargo delivery. Changes in ganglioside expression during development, aging, or pathological conditions could similarly influence EV biology by affecting their interactions with recipient cells.

A notable finding in our study with implications for cancer therapy is the ability of GA1 to block EV production in neuroblastoma cells. Tumor-derived EVs play critical roles in tumor progression, metastasis, and immune evasion (*98*, *99*). Given these key roles and that many tumors up-regulate EV biogenesis (*100*, *101*), inhibiting EV secretion with GA1 could provide a therapeutic approach, potentially disrupting multiple tumor-supporting processes.

Overall, our study demonstrates that gangliosides, both endogenous and exogenously applied, are potent modulators of EV secretion across multiple cell types. By elucidating their role in EV biogenesis, we provide insight into previously unreported pathogenic mechanisms in diseases characterized by ganglioside deficiency and into how GM1 may exert its mHTT-lowering and neuroprotective effects. Future work should address the precise molecular interactions by which gangliosides influence EV biogenesis and cargo loading to harness these effects to improve proteostasis and other EV-dependent processes.

## METHODS

### Chemicals, reagents, and materials

Purified porcine GM1 was provided by TRB Chemedica Int., Switzerland. Purified bovine GM3, GD3, C18 LacCer, and C18 GlcCer were purchased from Avanti Polar Lipids Inc., USA. Purified bovine GM2, GD2, and GQ1b were purchased from Cayman Chemical, USA. Purified GD1a, GD1b, and GT1b, semisynthetic asialo-GM1 and GM1 pentasaccharide were purchased from Enzo Life Sciences, USA. Genz-123346 was purchased from Toronto Research Chemicals, Canada. Vybrant DiI and Vybrant DiD Cell-Labeling solutions, mouse laminin, and Dulbecco’s modified Eagle’s medium (DMEM)/F12-Hepes (phenol red–free) were purchased from Thermo Fisher Scientific, USA. l-glutamine, sodium pyruvate, Geneticin, N-2 supplement, B27 supplement, GlutaMAX supplement, Opti-MEM I reduced serum media (with phenol red), Opti-MEM I reduced serum media (phenol red–free), DMEM–high glucose (phenol red–free), minimum essential medium (MEM) (with phenol red), MEM (phenol red–free), MEM nonessential amino acids (NEAA) solution, Hanks’ balanced salt solution (HBSS), Neurobasal-A medium (phenol red–free and with phenol red), phosphate-buffered saline (PBS, without calcium and magnesium), MEM NEAA, Essential 8 medium, KnockOut DMEM, StemPro Accutase, and horse serum were purchased from Gibco, Thermo Fisher Scientific, USA. Cell-culture grade dimethyl sulfoxide (DMSO), Tween-80, fetal bovine serum (FBS), EDTA, EGTA, MG132, bovine serum albumin (BSA), BSA free of fatty acids, and Amicon Ultra-15 Centrifugal Filters (10,000 or 100,000 MWCO) were purchased from Sigma-Aldrich, USA. Doxycycline hyclate was purchased from either Gibco, Thermo Fisher Scientific, USA (for Tau-inducible cells) or Sigma-Aldrich, USA (for iPSCs). Recombinant human Neurotrophin-3 and recombinant human brain–derived neurotrophic factor (BDNF) were purchased from Peprotech. Y-27632 dihydrochloride was purchased from MedChemExpress. DMEM–high glucose (with phenol red), cell-culture grade Dulbecco’s modified PBS (DPBS), and penicillin-streptomycin were purchased from HyClone, GE Healthcare, USA. Complete Protease inhibitor cocktail and PhosStop phosphatase inhibitor cocktail were purchased from Roche, Switzerland. IGEPAL CA-630 was from Sigma-Aldrich, USA. Nitrocellulose membranes were purchased from Bio-Rad, USA, while polyvinylidene difluoride (PVDF) membranes (Immobilon-FL) were from Merck Millipore, USA. Prelubricated microcentrifuge tubes (CoStar, Corning Life Sciences, USA) were used for the isolation and analysis of EVs. All polystyrene and polypropylene tubes were from Falcon, Thermo Fisher Scientific, USA. qEV Original 70-nm columns were purchased from IZON Science Ltd., USA. ExoView human tetraspanin plasma Kits were purchased from Unchained Laboratories, USA.

### Cell models

#### Mouse neuroblastoma Neuro2a cells

N2a [American Type Culture Collection (ATCC) CCL-131] were donated by S. Kar (University of Alberta, Canada). N2a cells were cultured in DMEM:Opti-MEM I (1:1) supplemented with 10% heat-inactivated FBS, 2 mM l-glutamine, and sodium pyruvate (0.11 g/liter, N2a growth medium) and maintained at 37°C with 5% CO_2_.

#### *Generation of N2a cells stably expressing mHTT-EGFP or* α*-synuclein (A53T)*

N2a cells were stably transfected with the cDNA for mHTT Exon 1 (containing 97 glutamines) tagged with EGFP [HTT-Exon1 (97Q) donated by J. Braun (University of Calgary, Canada)], or α-synuclein (A53T) tagged with EGFP purchased from Addgene (plasmid #40823). Cell transfection was performed using Lipofectamine 3000 reagent (Thermo Fisher Scientific, USA). Geneticin (800 μg/ml) was added to the medium to select stably transfected cells. GFP-fluorescent cells were further sorted into 96-well plates using FACS Aria III cell sorter (BD Biosciences, USA), at the Faculty of Medicine and Dentistry Flow Cytometry Facility, to select stably transfected clones. Transgene expression in selected clones was confirmed by immunoblotting. Stably expressing cell clones, referred to as N2a 97Q or N2a α-synuclein (A53T), were maintained in N2a growth media with Geneticin (400 μg/ml) at 37°C with 5% CO_2_. Geneticin was removed from the culture media one passage before performing experiments. Cells were routinely checked for GFP fluorescence by microscopy and were periodically resorted by fluorescence-activated cell sorting when the percentage of fluorescent cells fell below 80%.

#### *N2a* ΔB4galnt1

N2a Δ*B4galnt1* were obtained by deletion of *B4galnt1* from WT N2a cells using CRISPR-Cas9 as previously described (*68*) and maintained in N2a growth medium at 37°C with 5% CO_2_.

#### HeLa cells

HeLa cells (ATCC CCL-2) were donated by I. Swie Goping (University of Alberta, Canada) and grown in DMEM supplemented with 10% heat-inactivated FBS, 2 mM l-glutamine, and sodium pyruvate (0.11 g/liter, HeLa growth medium), at 37°C with 5% CO_2_.

#### Embryonic rat cortical neurons

Embryonic rat cortical neurons were isolated from E18 embryos and maintained in neuronal growth media (Neurobasal containing 200 μM Glutamax, penicillin (100 U/ml), streptomycin (100 μg/ml), and 1× B27 supplement) on plates coated with poly-l-lysine (molecular weight ≥ 300,000; Sigma Aldrich, USA). All procedures on animals were approved by the University of Alberta’s Animal Use Committee (Animal Use Protocol #336) and were in line with the Canadian Council on Animal Care. Briefly, pregnant dams (Sprague-Dawley) were subjected to isoflurane-anesthesia followed by decapitation. Thereafter, the uterus was transferred to a petri dish on ice, the embryos were isolated and decapitated, and the brains were dissected under a microscope. Dissected cortical tissue was resuspended in 0.25% trypsin-EDTA (Thermo Fisher Scientific, USA) in a 50-ml polystyrene tube and incubated for 20 min at 37°C in a water bath. The digested tissue was centrifuged at 300*g* for 2 min, the supernatant discarded, and the pellet resuspended in trituration media (DMEM +10% heat-inactivated FBS + 1% penicillin-streptomycin). Thereafter, the tissue was passed 10 times through a fire-polished glass Pasteur pipette and the supernatant obtained after tissue sedimentation was transferred to a new polystyrene tube. Trituration was performed three times in total. The cell suspension was then filtered through a 40-μm pore-size nylon cell strainer (Thermo Fisher Scientific, USA). Cells were counted using a hemocytometer and plated at a density of 0.13 × 10^6^ cells/cm^2^ in trituration medium. After cells had attached (1.5 to 2 hours after seeding), the trituration medium was replaced with neuronal growth media. On 2 day(s) in vitro (DIV2), the cells were treated with cytosine arabinoside (3 μM, Sigma-Aldrich, USA) for 48 hours to block the proliferation of glial cells and reduce their presence to less than 1% (*102*). Half of the medium was replaced with fresh medium every 4 days. Cultures were maintained at 37°C with 5% CO_2_. Experiments were performed on DIV15.

#### Primary human fibroblasts

Primary human fibroblasts purchased from the Coriell Institute for Medical Research, USA, were derived from 1 female and 2 male HD donors (HD11M, HD29F, and HD35M; Coriell codes GM04855, GM03621, and GM04208) and three age-matched healthy male donors (WT15M, WT33M, and WT36M; Coriell codes GM01869, AG08181, and AG07573). A complete characterization of these cells is available on the Coriell Institute for Medical Research website. Fibroblasts were maintained in MEM (with NEAAs) supplemented with 15% heat-inactivated FBS, 2 mM l-glutamine, and sodium pyruvate (0.11 g/liter) (fibroblast growth media), at 37°C with 5% CO_2_. For cell passaging and seeding, the cells were washed with HBSS (Ca^2+^/Mg^2+^-free) and then incubated with the same buffer containing 0.53 mM EDTA for 2 min at room temperature (r.t.) before cell trypsinization in 0.05% trypsin-EDTA (Corning Life Sciences, USA). The cells were passed before they could reach 80% confluence as cellular ganglioside levels may change upon reaching confluence (*103*).

#### Differentiation of human iPSCs into i^3^Neurons

Human iPSCs (on a male WTC11 background) expressing mNGN2 under the control of a doxycycline inducible promoter (*104*) were cultured on plates coated with Matrigel in KnockOut DMEM, grown in Essential 8 medium, and maintained at 37°C with 5% CO_2_. The medium was changed every day, and the cells were split once they reached 80% confluency using Accutase. To generate glutamatergic neurons (i^3^Neurons), iPSCs were differentiated following a previously described protocol (*104*). Briefly, i^3^Neurons iPSCs were predifferentiated in growth medium composed of KO-DMEM supplemented with NEAA, mouse laminin (1 μg/ml), Y-27632–ROCK inhibitor (3.2 mg/ml), BDNF (10 ng/ml), NT3 (10 ng/ml), N-2 supplement, and doxycycline (2 μg/ml) for 3 days, with daily medium changes and ROCK inhibitor removed from day 2. Predifferentiated cells were then dissociated using StemPro Accutase and seeded in plates coated with high molecular weight poly-d-lysine in differentiation medium composed of DMEM/F12-Hepes:Neurobasal-A medium (1:1), supplemented with NEAA, mouse laminin (1 μg/ml), BDNF (10 ng/ml), NT3 (10 ng/ml), GlutaMax, and 0.5× N-2 and 0.5× B27 supplement. Half of the medium was replaced on day 7. On days 14 and 21, half the medium was replaced with double the volume, and then one-third of the medium was replaced weekly until i^3^Neurons were used on day 56.

#### PC12 14A2.5

PC12 14A2.5 cells expressing mHTT(exon 1)–EGFP were previously described (*70*). They were maintained in DMEM supplemented with 5% heat-inactivated FBS, 10% heat-inactivated horse serum, 2 mM l-glutamine, and sodium pyruvate (0.11 g/liter), at 37°C with 5% CO_2_. Cell passaging was performed by trypsinization in 0.05% trypsin-EDTA (Corning Life Sciences, USA). Expression of mHTT-EGFP was induced by incubating cells with 5 μM ponasterone A for 6 or 72 hours, as indicated, and as previously described (*70*).

#### STHDh 111/111

STHDh 111/111 conditionally immortalized knock-in striatal cells expressing full-length mHTT were maintained in DMEM supplemented with 10% heat-inactivated FBS, 2 mM l-glutamine, and sodium pyruvate (0.11 g/liter) at 33°C with 5% CO_2_, as previously described (*71*) .

#### Inducible HEK293 cells expressing WT, N297K, or P301L tau

Inducible HEK293 cells expressing WT, N297K, or P301L tau were engineered using the Flp-In T-REx HEK293 cell line (Thermo Fisher Scientific, USA). These cell lines are characterized by targeted integration of a construct for GFP-0N4R tau (WT or mutant) under the control of a doxycycline-inducible promoter into a single FRT integration site within the cell genome. The generation of GFP-0N4R P301L tau cell line has been previously described (*105*). The N279K and WT GFP-0N4R tau cell lines were generated by the same procedure. Tau HEK293 cells were cultured in DMEM high glucose supplemented with 10% FBS, glutamine (2 mM), sodium pyruvate (1 mM), and Hygromycin B (0.15 mg/ml), and maintained at 37°C with 5% CO_2_. The cells were treated with doxycycline (10 ng/ml) for 72 hours to induce WT or mutant GFP-0N4R tau expression.

### In-cell labeling for EV detection

Indirect fluorescent labeling of EVs was performed as previously described (*53*) to detect and quantitate EVs by fluorometry or IFC. Cell membranes (including EV membranes) were labeled with the lipophilic membrane stain DiI (λ_Ex_/λ_Em_ = 549/565 nm) or DiD (4-chlorobenzenesulfonate salt; λ_Ex_/λ_Em_ = 644/665 nm), according to the manufacturer’s instructions (Invitrogen, Thermo Fisher Scientific, USA). Briefly, 5 μl of the dye solution (1 mM) was added to each 1 ml of cell suspension containing 1 × 10^6^ cells in serum-free media and incubated for 8 to 20 min in the dark at 37°C in a polypropylene tube. The suspension was then centrifuged at 400*g* for 5 min at r.t., and the stained cell pellet was washed three times in serum-containing growth medium to remove any unbound dye. The cells were then resuspended in growth medium and seeded in 100-mm dishes. The seeding densities were determined such that the confluence of the cells at the time of harvesting was approximately 80%.

### Preparation of EV collection medium

The medium used for EV collection from cells was either serum-free medium supplemented with heat-inactivated EV-depleted FBS (EVD complete medium) or phenol red–free, serum-free medium supplemented with N-2 supplement (SFM + N-2). To prepare EV-depleted FBS, heat-inactivated FBS was transferred to 26.3-ml thickwall polycarbonate tubes (Beckman Coulter, USA) and centrifuged at 100,000*g* for 16 hours at 4°C, using a Type 70 Ti fixed-angle rotor (Beckman Coulter, USA) in an Optima L-70 ultracentrifuge (Beckman Coulter, USA) (*106*). The supernatant was filter-sterilized with a 0.22-μm PVDF filter (Millipore, USA) and stored at −20°C until further use. Collection medium containing N-2 supplement was filtered through a 0.1-μm PVDF filter (Millipore, USA) before use.

### Cell treatment with glycosphingolipids

Porcine ganglioside GM1 was dissolved in cell-culture grade DPBS or DMSO to a stock concentration of 10 mM. Bovine gangliosides GM2, GD3, GD2, GD1a, GD1b, GT1b, GQ1b, and the GM1 pentasaccharide were dissolved in cell-culture grade DPBS to a stock concentration of 10 mM. GlcCer, GA1, and GM3 were dissolved in cell-culture grade DMSO to a stock concentration of 10 mM. To completely dissolve LacCer in cell-culture grade DMSO, the 10 mM stock solution was warmed to 40°C for 10 min, sonicated in a water bath sonicator for 5 min, and kept at 40°C until use. Before dilution for cell treatment, the LacCer solution was sonicated as above and heated for 10 min at 40°C. Gangliosides and glycosphingolipids were added to cells in growth media or SFM + N-2 upon cell seeding or after cell attachment to the growth plate (2 to 4 hours after seeding) at 1 to 50 μM concentration, as indicated. Where indicated, gangliosides were washed off with two washes with HBSS (with Ca^2+^/Mg^2+^) supplemented with 0.2% essentially fatty acid-free BSA, followed by two washes with HBSS (with Ca^2+^/Mg^2+^).

### Inhibition of ganglioside synthesis with Genz-123346

Genz-123346 was solubilized in 100% cell-culture grade DMSO to a stock concentration of 10 mM and stored at −20°C until use. It was added to cells in growth media at the time of cell seeding to a final concentration of 1 μM (the final concentration of DMSO was 0.01%). DMSO at the final concentration of 0.01% was added to vehicle control groups. After 24 hours of treatment, the cells were washed with HBSS (with Ca^2+^/Mg^2+^) and the treatment was replenished in EV collection media.

### EV collection and isolation

After cell treatment, the medium was replaced with EV collection medium and allowed to be conditioned by cells for 16 to 24 hours to collect EVs. To remove loose cells, apoptotic bodies, and cell debris, the conditioned medium was collected in polypropylene tubes and centrifuged at 2000*g* for 10 min at 4°C in an Eppendorf Centrifuge 5810 R, using an A-4-62 swinging bucket rotor. The supernatant retained for analysis is hereafter referred to as the CCM. To avoid EV loss, where possible, prelubricated low protein-binding microcentrifuge tubes were used in the subsequent steps of EV isolation and analysis. Depending on the experiment, ultracentrifugation or ultrafiltration followed by SEC were used to isolate EVs.

#### Ultracentrifugation

The CCM was ultracentrifuged at 100,000*g* for 90 min at 4°C in a 10.4-ml thickwall polycarbonate tube (Beckman Coulter, USA) using an MLA-55 fixed-angle rotor (Beckman Coulter, USA) in an Optima MAX-XP tabletop ultracentrifuge (Beckman Coulter, USA). The EV pellet was washed by resuspension in 0.6 ml of DPBS in a 1-ml open-top thickwall polycarbonate tube (Beckman Coulter, USA) and ultracentrifugation at 100,000*g* for 90 min at 4°C using an MLA-130 fixed-angle rotor (Beckman Coulter, USA). The final EV pellet was resuspended in 100 μl of DPBS.

#### Ultrafiltration and SEC

Large volumes (>50 ml) of CCM were concentrated using Amicon Ultra-15 Centrifugal Filters (100,000 MWCO) (Millipore Sigma, USA) according to the manufacturer’s instructions. To minimize EV loss due to protein binding to the filter membranes, the latter were pretreated with 5% Tween-80 (*107*) in DPBS and centrifuged at 2608*g* (Eppendorf Centrifuge 5810 R, A-4-62 swinging bucket rotor) for 10 min at 4°C, followed by three washes in DPBS for 5 min at the same centrifugal speed. Once blocked, filter membranes were kept in DPBS until use to prevent drying. The CCM was concentrated using these filters by centrifugation at 2608*g* at 4°C. The time required to concentrate the material depended on the initial CCM volume, the final required retentate volume, and the composition of the media being filtered. The concentrated CCM (retentate) was immediately transferred to a new tube, and the filter membrane was washed three times with DPBS to collect any loosely adherent material. EVs in the retentate were isolated by qEV Original 70-nm size exclusion columns. SEC columns were stored in DPBS containing 0.05% sodium azide at 4°C and used according to the manufacturer’s instructions. After column equilibration at r.t. and the initial flush of 10 ml of DPBS, 0.5 ml of the retentate obtained by ultrafiltration was loaded onto the column and eluted with DPBS in 0.5-ml fractions. The presence of EVs in the various fractions was determined by measuring DiI (λ_Ex_/λ_Em_ = 540/580 nm) or DiD (λ_Ex_/λ_Em_ = 644/674 nm) fluorescence in each fraction. The protein content of each fraction was determined by measuring the absorbance at 280 nm using Nanodrop 2000c (Thermo Fisher Scientific, USA). Fractions enriched with EVs were pooled and concentrated using Amicon Ultra 2-ml filters (10,000 MWCO) that were pretreated with Tween-80 as described above. EV fractions were concentrated by centrifugation at 2608*g* (with an A-4-62 swinging bucket rotor in an Eppendorf Centrifuge 5810 R) at 4°C.

### Storage and handling of EV samples

All EV samples were kept on ice and analyzed immediately after isolation for the determination of their number, size distribution, and phenotype by IFC, ExoView, NTA, and DLS. For other applications, EV samples were stored at −80°C until use. For uptake studies, EVs were stored overnight at 4°C before IFC quantification and incubation with recipient cells. Repeated freeze-thaw cycles were avoided. To limit EV particle damage, EV samples were mixed by pipetting or by tube inversion and never vortexed.

### EV uptake by N2a cells

N2a cells were stained with DiD as described above and seeded in growth medium [DMEM supplemented with 10% heat-inactivated FBS, 2 mM l-glutamine, and sodium pyruvate (0.11 g/liter)]. Following attachment, the cells were treated with 50 μM GM1 for 18 hours. GM1 was washed off as described above, and cells were incubated in collection medium [phenol red–free DMEM supplemented with 2 mM l-glutamine and sodium pyruvate (0.11 g/liter)] for 24 hours. EVs secreted by control and GM1-treated cells were isolated by SEC and quantified by IFC.

Recipient unlabeled N2a cells were plated at a density of 1 × 10^5^ per well in 24-well plates (Fisher Scientific, USA) and incubated for 18 hours with GM1 or vehicle in growth medium before exposure to EVs. After washing, the cells were incubated with 1 × 10^7^ DiD-labeled EVs (prepared as indicated above) diluted in 200 μl of collection medium [phenol red–free DMEM:Opti-MEM I (1:1) supplemented with N-2 supplement, 2 mM l-glutamine, and sodium pyruvate (0.11 g/liter)] for 10 to 180 min. The cells were then trypsinized to lift them from the culture plates, centrifuged at 300*g* for 5 min, and resuspended in 80 μl of 2% paraformaldehyde (Electron Microscopy Sciences, USA). Last, the cells were transferred to a U-bottom 96-well plate for uptake analysis by IFC.

### IFC analysis

IFC was used to determine EV number, detect mHTT-GFP within EVs, and measure EV uptake by N2a cells. EV analysis by IFC was performed according to an established protocol (*53*) using an Amnis ImageStream mkII instrument, at the Flow Cytometry Core Facility of the Faculty of Medicine and Dentistry at the University of Alberta. All data were acquired with fluorescence lasers set at maximum power, with 60× magnification, and the lowest flow rate. Data were acquired after 1 min of sample loading into the instrument to allow for flow stabilization. The DiI or DiD signal associated with EVs was acquired using a 560- to 595-nm filter or a 642- to 745-nm filter, respectively. A 435- to 480-nm filter was used for bright-field imaging, and a 745- to 780-nm filter was used for side scatter (SSC) measurements. GFP fluorescence in EVs carrying mHTT-GFP was detected using a 480- to 560-nm filter. DPBS (pH 7.4) (BioSure Sheath Solution, BioSure, UK) was used as sheath fluid. If required, the samples were diluted with DPBS to avoid coincidence events. For most experiments, 3 to 10 technical replicates were acquired for each sample and data were averaged. In each experiment, 10,000 events were acquired for the control sample, gating for events with a SSC lower than that of the SpeedBeads (Amnis SpeedBead Kit for ImageStream, Luminex Corporation, USA). All other samples were acquired for the same duration as the control. To confirm that the particles detected were membrane-bound EVs, each sample was reanalyzed after incubation with 0.05% NP-40 (IGEPAL CA-630, filtered through 0.1-μm PVDF filter) for 30 min at r.t., to solubilize all membrane-bound particles. Data were analyzed using IDEAS software v6.2. Any speed beads that may have been captured during the acquisition were gated out, and masks were made to identify positive particles as described below:

- DiI: Intensity(M03,Ch03_DiI, 50-4095)

- DiD: Intensity(M11,Ch11_DiD, 60-4095)

As DiI or DiD were the fluorescent pan-EV markers, the spot count feature was added to their analysis to detect single particles.

Analysis of EV uptake was performed in the autosampler mode using samples loaded into a U-bottom 96-well plate and covered with X-Pierce film (Excel Scientific Inc., USA) to reduce evaporation during sample acquisition. Data were acquired as described above with 40× magnification. Acquisition gates were set to acquire 8000 in-focus, round, and single cells per sample. Events with a gradient root mean square of the bright-field image above 50 were gated as in-focus. Bright-field images with high aspect ratio and low area were gated to identify the single, round cells. To exclude any DiD signal from EVs adhering to the surface of cells rather than internalized by the cells, an adaptive erode mask with an erosion coefficient of 85 was applied to the bright-field images. A histogram of the intensity of DiD within the adaptive erode mask was generated and used to gate for DiD-positive events and calculate mean fluorescent intensity of the DiD-positive cells. Data were analyzed using IDEAS software v6.4.

### Fluorometry

DiI or DiD fluorescence was measured in cell lysates and EV fractions using a SpectraMax i3x multimode microplate reader (Molecular Devices, USA). Ex/Em for DiI is 540/580 nm while that for DiD is 644/674 nm. The bandwidth of all excitation and emission wavelengths was set to 15 nm. Fluorescence was measured in 96-well black-bottom plates by well-scan reading.

### CytoFlex EV analysis

Unlabeled EV samples (SEC-isolated or CCM) were analyzed using the CytoFLEX S (Beckman Coulter, USA) instrument at the Flow Cytometry Core Facility of the Faculty of Medicine and Dentistry at the University of Alberta. Samples were diluted 1/500 (SEC-isolated) or 1/10 (CCM) with 0.1 μm of filtered DPBS. Following flow stabilization, each sample was recorded for 60 s on slow speed and triggered with the 405-nm violet SSC (V-SSC) laser with a detection threshold of 1750. The V-SSC gain was set to 200. Data analysis was performed using CytExpert software. V-SSC^+^ events that were resolved from the electronic noise were gated on the basis of DPBS for SEC-isolated samples or unconditioned collection medium diluted 1/10 with DPBS for CCM samples.

### Nanoparticle tracking analysis

EV number and size distribution were determined using the NanoSight LM10 system (405-nm laser, software version 3.3, Sample Assistant Dev Build 3.3.203) (NanoSight, UK). Instrument sizing was checked using 200-nm polystyrene beads (Nanosphere, USA) resuspended in 10 mM potassium chloride (KCl). CCM samples were diluted with 0.1 μm of filtered PBS (Gibco, Thermo Fisher Scientific, USA) to acquire 20 to 90 particles per frame. Three hundred microliters of the sample was injected into the NanoSight chamber with a 1-ml syringe. Five serial measurements were taken for each sample, and each measurement was taken for 1 min. PBS was injected into the chamber to wash the system in between samples.

### Dynamic light scattering

Freshly prepared retentate obtained by ultrafiltration of the CCM was used for DLS measurements to determine EV size distribution. An aliquot of the retentate corresponding to 350 μg of proteins (measured by BCA protein assay) was subjected to asymmetric flow field-flow fractionation on an AF2000 Postnova system, using DPBS (pH 7.4) as running buffer. Samples were focused for 5 min before elution at a flow rate of 0.5 ml/min. A slot pump was run at 0.3 ml/min to concentrate the samples before passing them through the detectors. Fractions of 0.2 ml were collected and analyzed by an in-line DAWN HELEOS II detector (Wyatt Technology) and using ASTRA software v6.1.

### ExoView EV capture and analysis

Diluted CCM was incubated at r.t. overnight on ExoView microchips carrying immobilized antibodies against the tetraspanins CD9, CD63, and CD81. The next day, the chips were washed using the ExoView C100 plate washer and incubated with fluorescent anti-tetraspanin antibodies. The EVs captured on the chip were sized by single-particle interferometric reflectance imaging (SP-IRIS) and quantified using an ExoView R200 instrument. Data were analyzed using ExoView Analyzer 3.2.1.

### Protein quantification

Protein concentration in cell lysates and EV samples was measured with a Pierce BCA protein assay or with a Pierce Enhanced BCA protein assay (Thermo Fisher Scientific, USA), respectively, according to the manufacturer’s instructions. All samples for protein measurements were loaded in triplicates. The absorbance of each sample was measured by endpoint reading at 562 nm, using a SpectraMax i3x multi-mode microplate reader (Molecular Devices, USA).

### Detection and quantitation of ganglioside GM1

Relative quantification of GM1 in cell lysates and EV samples was performed by dot-blotting. Cells lysed in NP-40 lysis buffer [20 mM tris-HCl (pH 7.4), 1% NP-40 v/v, 1 mM EDTA, 1 mM EGTA, 50 μM MG132, and 1× protease/phosphatase inhibitor cocktail] were passed through a 27-G needle 10 times, incubated on ice for 30 min, and then sonicated at intensity 2.0 for 10 s using a Sonic Dismembrator Model 100 (Fisher Scientific, USA), before sample immobilization onto a 0.45-μm pore size nitrocellulose membrane (Millipore, USA) using a 96-well Bio-Dot apparatus (Bio-Rad, USA). EV samples were either sonicated at intensity 2.0 for 10 s or lysed with 0.01% NP-40. One microgram of protein from cell lysates was loaded in each well, in triplicates. EV samples were loaded in duplicates or triplicates. Membranes were blocked for 1 hour with Odyssey blocking buffer or Intercept blocking buffer (LiCor Biosciences, USA), and incubated overnight at 4°C or for 2 hour at r.t. with biotinylated cholera toxin B (0.25 μg/ml, Thermo Fisher Scientific, USA) diluted in Odyssey or Intercept blocking buffer to detect GM1, and with anti–α-tubulin antibodies (2125, Cell Signaling Technology, USA) to control for equal loading of cell lysates. Membranes were then washed with TBS-T [20 mM tris-HCl an d 137 mM NaCl (pH 7.4 to 7.6) containing 0.1% Tween-20] and incubated with IRDye800-conjugated Streptavidin (LiCor Biosciences, USA) diluted 1:5000, and the appropriate fluorescent secondary antibody diluted 1:10,000, with Odyssey or Intercept blocking buffer:TBS-T (1:3) for 1 hour at r.t. Last, membranes were washed once with TBS-T and once more with TBS before scanning in an Odyssey near-infrared scanner (LiCor Biosciences, USA). Signal was quantified using Odyssey Application Software v3.0 (LiCor Biosciences, USA).

### Immunoblotting

Cell lysates (30 to 60 μg of proteins) and equal fractions of EV samples isolated by ultracentrifugation or by SEC (not exceeding 60 μg of total EV proteins) were denatured in sample loading buffer [62.5 mM tris-HCl (pH 6.8), 2% SDS, 10% glycerol, 5% 2-mercaptoethanol, and bromophenol blue (6.25 mg/ml)] for 5 min at 95°C. Samples were loaded onto 4 to 20% SDS–polyacrylamide gel electrophoresis (SDS-PAGE) gradient gels and run in a Mini-PROTEAN II electrophoresis chamber (Bio-Rad, USA). Proteins were transferred to a 0.45-μm PVDF membrane in Towbin buffer (25 mM tris-HCl, 192 mM glycine, and 20% methanol v/v) using a Mini Trans-Blot apparatus (Bio-Rad, USA). For full-length huntingtin immunoblots, samples were loaded onto 4 to 12% SDS-PAGE gels and run as above, and protein transfer was performed using a modified Towbin buffer [25 mM tris-HCl, 192 mM glycine, 16% methanol v/v, and 0.5% SDS]. PVDF membranes were blocked for 1 hour with 5% BSA in TBS-T, Odyssey, or Intercept blocking buffer (LiCor Biosciences, USA) and then incubated with primary antibodies for 2 hours at r.t. or overnight at 4°C. The complete list of antibodies used can be found in table S1. The membranes were then washed with TBS-T and incubated with the appropriate fluorescent secondary antibodies (LiCor BioSciences, USA) diluted in blocking buffer:TBS-T (1:3) for 1 hour at r.t. After washing with TBS-T and TBS, the membranes were scanned in an Odyssey or Odyssey M near-infrared scanner (LiCor Biosciences, USA) and signal was quantified using Odyssey Application Software v3.0 (LiCor Biosciences, USA), Empiria Studio v3.1 or using ImageJ software (NIH, USA). Signal from cell lysates was normalized over Revert total protein stain (LiCor Biosciences, USA) or tubulin signal as indicated.

### ELISA for EGFP-conjugated proteins

Levels of mHTT-EGFP, WT, or mutant tau-EGFP or A53T α-synuclein–EGFP in EVs isolated from the CCM by SEC were measured by the GFP SimpleStep ELISA kit (Abcam, USA), following the manufacturer’s instructions. All materials and reagents were equilibrated to r.t. before use. The endpoint reading was recorded by measuring absorbance at 450 nm using SpectraMax i3x multi-mode microplate reader (Molecular Devices, USA). Data were normalized over total cellular protein content.

### Proteinase K protection assay

Two million N2a 97Q cells were seeded in each of 12 100-mm dishes. After 2 hours to allow for cell attachment, the cells were incubated with GM1 (50 μM) in SFM + N-2 for 6 hours or left untreated (control). GM1 was washed off, and EVs were collected in the conditioned medium for 16 hours and isolated by SEC as described above. Proteinase K (Sigma-Aldrich, USA) was solubilized in 25 mM tris-HCL (pH 8.0) containing 5 mM CaCl_2_ (buffer A) to a concentration of 0.1 mg/ml. For each experimental group, three EV aliquots (20 μg each) were freshly prepared and treated as follows: aliquot 1, incubation with 2.0 μg of proteinase K in buffer A for 1 hour at 37°C, to digest accessible proteins on the EV surface, followed by enzyme inactivation at 95°C for 10 min and sample incubation with 0.1% saponin (Sigma-Aldrich, USA) for 20 min at 37°C to permeabilize EV membranes and allow for the detection of EV luminal proteins; aliquot 2, incubation with 0.1% saponin for 20 min at 37°C to permeabilize EV membranes and allow access to the luminal content, followed by treatment with 2.0 μg of proteinase K and heat inactivation. This results in the digestion of all EV proteins (luminal and membrane associated); aliquot 3: incubation with 0.1% saponin for 20 min at 37°C to permeabilize EV membranes, followed by incubation in buffer A (equivalent volume to the proteinase K–treated samples) for 1 hour at 37°C and heating at 95°C for 10 min.

Following treatment, NP-40 was added to all samples at a final concentration of 0.05% and incubated for 30 min at r.t. to lyse EVs completely. Samples were spotted on a 0.45-μm pore size nitrocellulose membrane (Millipore, USA) using a 96-well Bio-Dot apparatus (Bio-Rad, USA), after dilution with DPBS to reduce the final concentration of NP-40 and saponin to no higher than 0.01 and 0.0167%, respectively, to avoid detergent interference with sample binding to the membrane. The membrane was blocked for 1 hour with Intercept blocking buffer (LiCor Biosciences, USA) and incubated with anti-Alix (611621, BD Biosciences, USA) and anti-HTT N17 antibodies (a gift from R. Truant, McMaster University, Canada) overnight at 4°C. The membrane was then washed with TBS-T and incubated with fluorescent secondary antibodies (LiCor Biosciences, USA) for 1 hour at r.t. After washing again with TBS-T and TBS, the membrane was scanned in an Odyssey near-infrared scanner (LiCor Biosciences, USA). Signal was quantified using ImageJ software (NIH, USA).

### Statistical analysis

All statistical analyses were performed in GraphPad Prism v10. Unless otherwise stated, two-tailed paired *t* test or ratio paired *t* test were used for pair-wise comparisons. One-way analysis of variance (ANOVA) with Tukey’s post hoc test or repeated measures one-way ANOVA with Holm-Šidák post hoc test were used to compare multiple groups. For comparing multiple groups with two or more independent variables, two-way ANOVA was used with Tukey’s post hoc test.
