# Supplementary material for: Gangliosides modulate the secretion of extracellular vesicles and their misfolded protein cargo
Source: Sci Adv. 2025 Sep 17;11(38):eady5212. doi: 10.1126/sciadv.ady5212 (PMC12442881; doi:10.1126/sciadv.ady5212)
Supplement: Supplementary file 1 — Figs. S1 to S6 Table S1 [file sciadv.ady5212_sm.pdf]

Science Advances
AAAS

Supplementary Materials for

Gangliosides modulate the secretion of extracellular vesicles and their misfolded protein cargo

John Monyror *et al.*

Corresponding author: Simonetta Sipione, ssipione@ualberta.ca; Elena Posse de Chaves, elena.chaves@ualberta.ca

Sci. Adv. **11**, eady5212 (2025)
DOI: 10.1126/sciadv.ady5212

This PDF file includes:

Figs. S1 to S6
Table S1

Figure S1

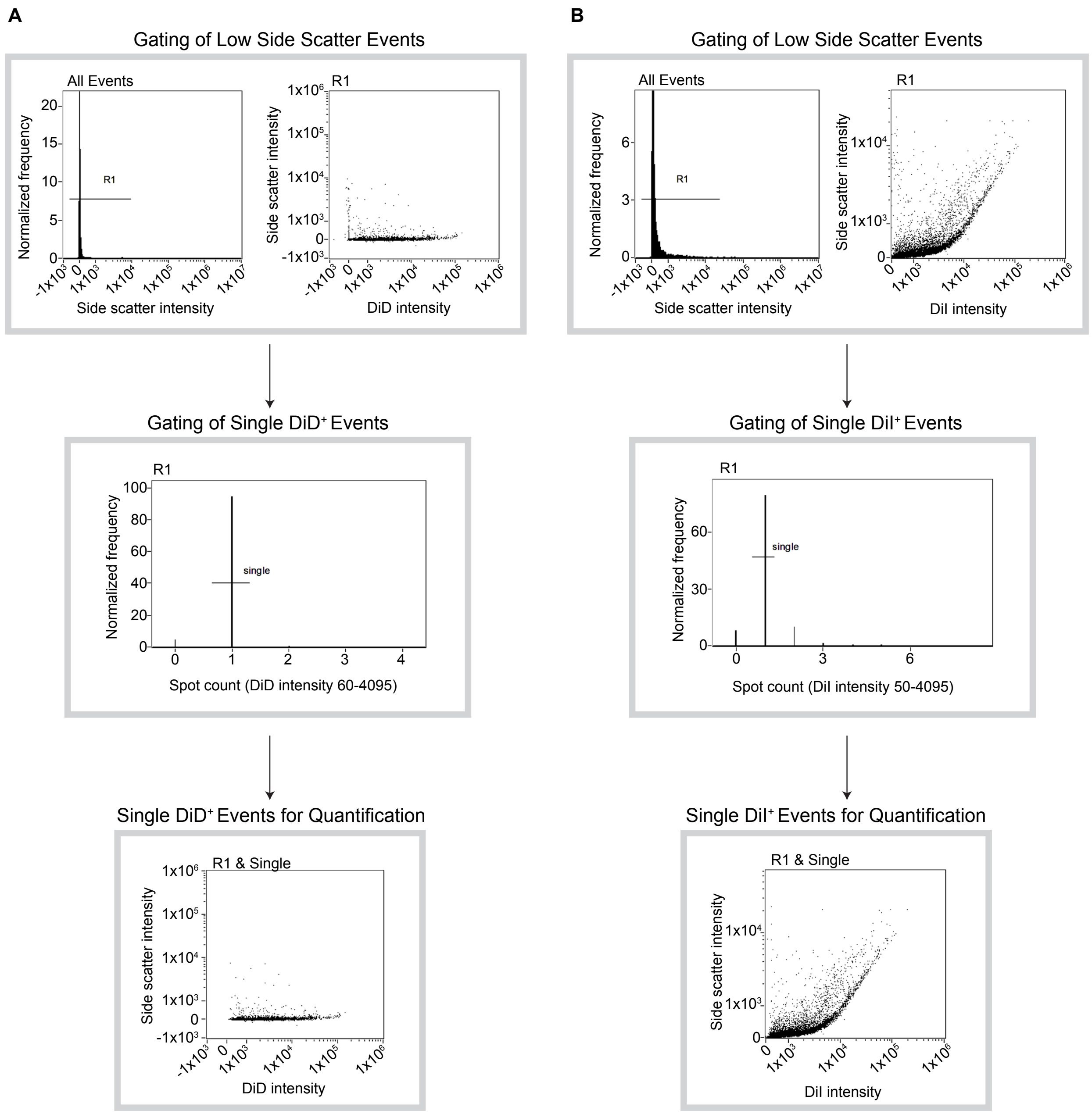
A
Gating of Low Side Scatter Events
All Events
Normalized frequency
Side scatter intensity
R1
Side scatter intensity
DiD intensity
Gating of Single DiD+ Events
R1
Normalized frequency
single
Spot count (DiD intensity 60-4095)
Single DiD+ Events for Quantification
R1 & Single
Side scatter intensity
DiD intensity
B
Gating of Low Side Scatter Events
All Events
Normalized frequency
Side scatter intensity
R1
Side scatter intensity
DiI intensity
Gating of Single DiI+ Events
R1
Normalized frequency
single
Spot count (DiI intensity 50-4095)
Single DiI+ Events for Quantification
R1 & Single
Side scatter intensity
DiI intensity

Fig. S1. Imaging flow cytometry gating strategy for DiD- and DiI-stained EVs.

Gating strategy for DiD^+-EVs (**A**) and DiI^+-EVs (**B**). Low side scatter events were gated to remove speedbeads ("R1" gate). An intensity mask was created to identify events with a fluorescent intensity above background, between 60 and 4095 for DiD^+-events or between 50 and 4095 for DiI^+-events. The spot count feature was used to identify and gate for single fluorescent events and quantify DiD^+- or DiI^+-EVs (R1 & single).

Figure S2

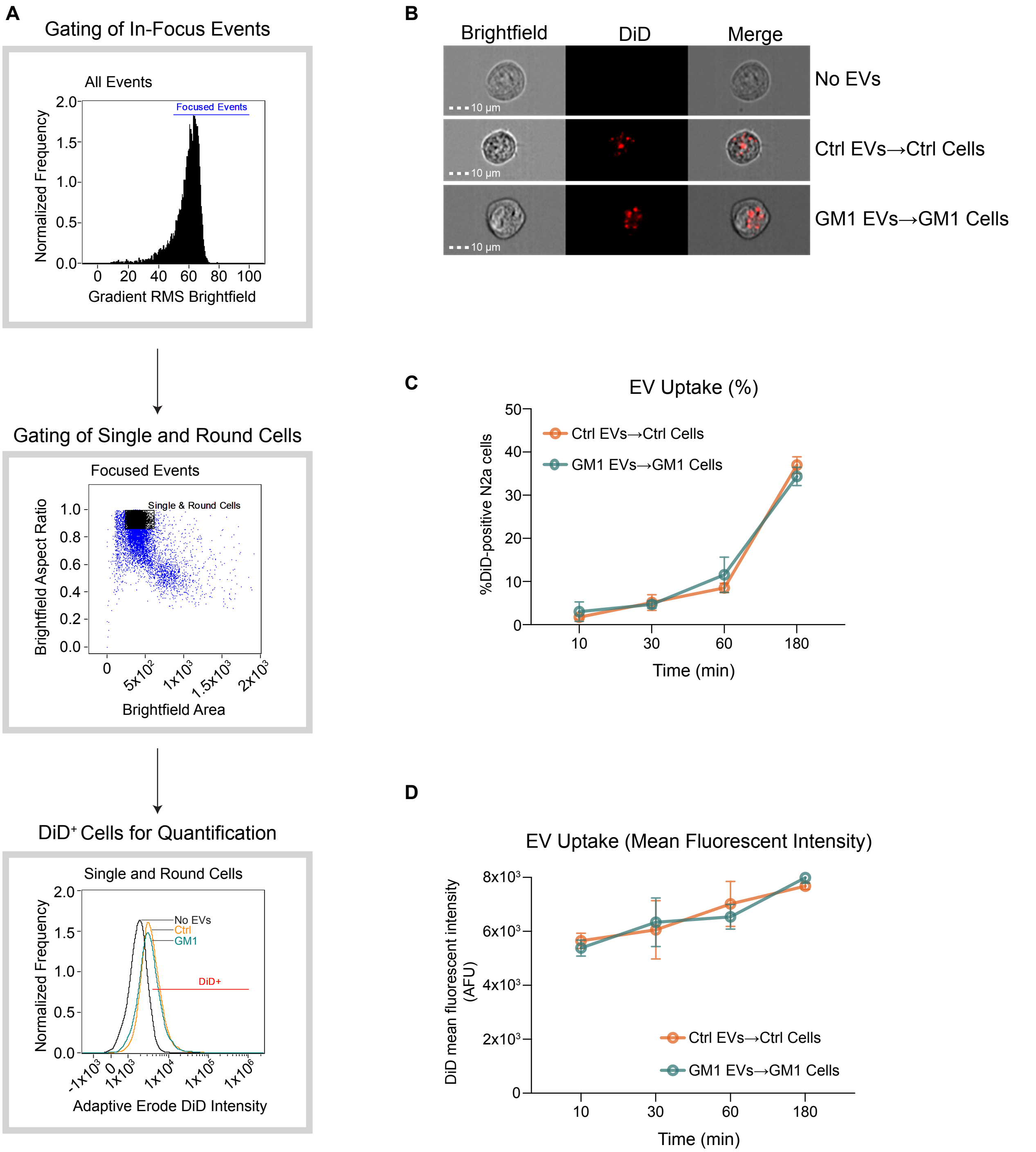
A
Gating of In-Focus Events
All Events
Focused Events
Normalized Frequency
2.0
1.5
1.0
0.5
0.0
0
20
40
60
80
100
Gradient RMS Brightfield
Gating of Single and Round Cells
Focused Events
Single & Round Cells
Brightfield Aspect Ratio
1.0
0.8
0.6
0.4
0.2
0.0
0
5x10²
1x10³
1.5x10³
2x10³
Brightfield Area
DiD⁺ Cells for Quantification
Single and Round Cells
Normalized Frequency
No EVs
Ctrl
GM1
DiD+
-1x10³
0
1x10³
1x10⁴
1x10⁵
1x10⁶
Adaptive Erode DiD Intensity
B
Brightfield
DiD
Merge
No EVs
Ctrl EVs→Ctrl Cells
GM1 EVs→GM1 Cells
10 μm
C
EV Uptake (%)
%DiD-positive N2a cells
50
40
30
20
10
0
Ctrl EVs→Ctrl Cells
GM1 EVs→GM1 Cells
10
30
60
180
Time (min)
D
EV Uptake (Mean Fluorescent Intensity)
DiD mean fluorescent intensity (AFU)
8x10³
6x10³
4x10³
2x10³
0
Ctrl EVs→Ctrl Cells
GM1 EVs→GM1 Cells
10
30
60
180
Time (min)

Fig. S2. Uptake of EVs by N2a Cells

(**A**) Gating strategy for EV uptake analysis. Only single, round cells in focus were gated for the analysis. DiD-positive cells were gated based on comparison with cells that did not receive labelled EVs (No EVs). Mean fluorescence intensity was obtained after applying a mask to exclude any fluorescence on the surface of cells.

(**B**) Representative brightfield, DiD, and merged images of cells after 180 minutes of incubation with or without EVs.

(**C**) Quantification of the percentage of cells with internalized EVs.

(**D**) Quantification of DiD mean fluorescent intensity in the DiD-positive cells.

Points represent means ± SD (n=3 technical replicates).

Figure S3

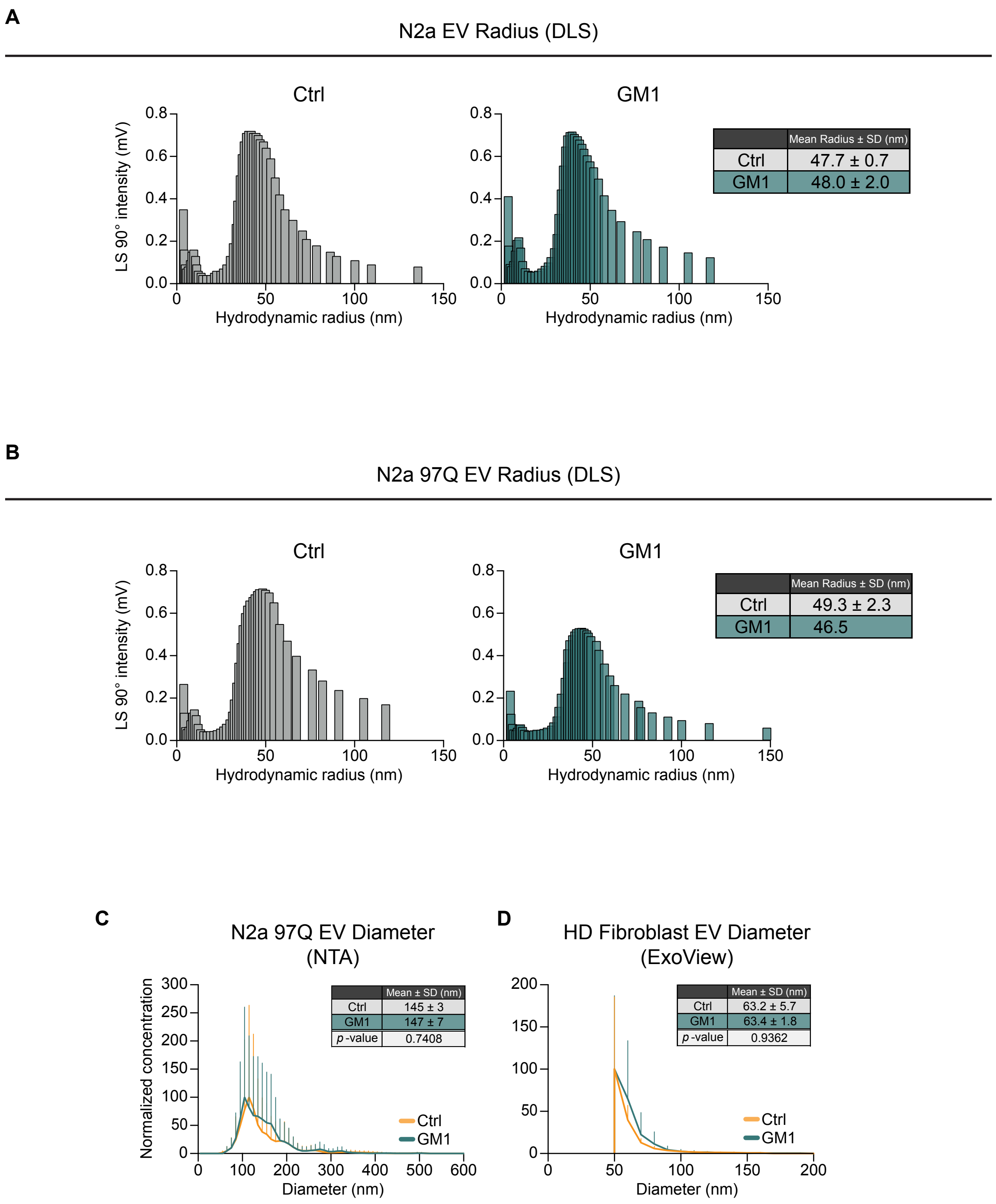

A
N2a EV Radius (DLS)
Ctrl
GM1
LS 90° intensity (mV)
0.0
0.2
0.4
0.6
0.8
0
50
100
150
Hydrodynamic radius (nm)
Mean Radius ± SD (nm)
Ctrl 47.7 ± 0.7
GM1 48.0 ± 2.0
B
N2a 97Q EV Radius (DLS)
Ctrl
GM1
LS 90° intensity (mV)
Hydrodynamic radius (nm)
Mean Radius ± SD (nm)
Ctrl 49.3 ± 2.3
GM1 46.5
C
N2a 97Q EV Diameter
(NTA)
Normalized concentration
0
50
100
150
200
250
300
Diameter (nm)
0
100
200
300
400
500
600
Mean ± SD (nm)
Ctrl 145 ± 3
GM1 147 ± 7
p -value 0.7408
Ctrl
GM1
D
HD Fibroblast EV Diameter
(ExoView)
0
50
100
150
200
Diameter (nm)
Mean ± SD (nm)
Ctrl 63.2 ± 5.7
GM1 63.4 ± 1.8
p -value 0.9362
Ctrl
GM1

Fig. S3. Sizing of EVs from cells treated with GM1.

(**A-B**) Representative histograms of the hydrodynamic radius distribution of EVs secreted by control and GM1-treated (50 μM) N2a cells (**A**) and control and GM1-treated N2a 97Q (**B**), as measured by dynamic light scattering (DLS). The tables show mean radius values ± SD for each group (n=1-2 experiments).

(**C**) Particle size distribution profile of EVs secreted by control and GM1-treated N2a 97Q and measured by nanoparticle tracking analysis (NTA). EV concentration is normalized to total cellular protein content. The table shows the mean particle diameter ± SD and *p*-values obtained by two-tailed paired *t*-test (n=3 experiments).

(**D**) Particle size distribution profile of EVs secreted by untreated and GM1-treated HD fibroblasts as measured by ExoView. EV counts are normalized to total cellular protein content. The table shows the mean particle diameter ± SD and *p*-values obtained by two-tailed paired *t*-test (n=3 biological replicates).

Figure S4

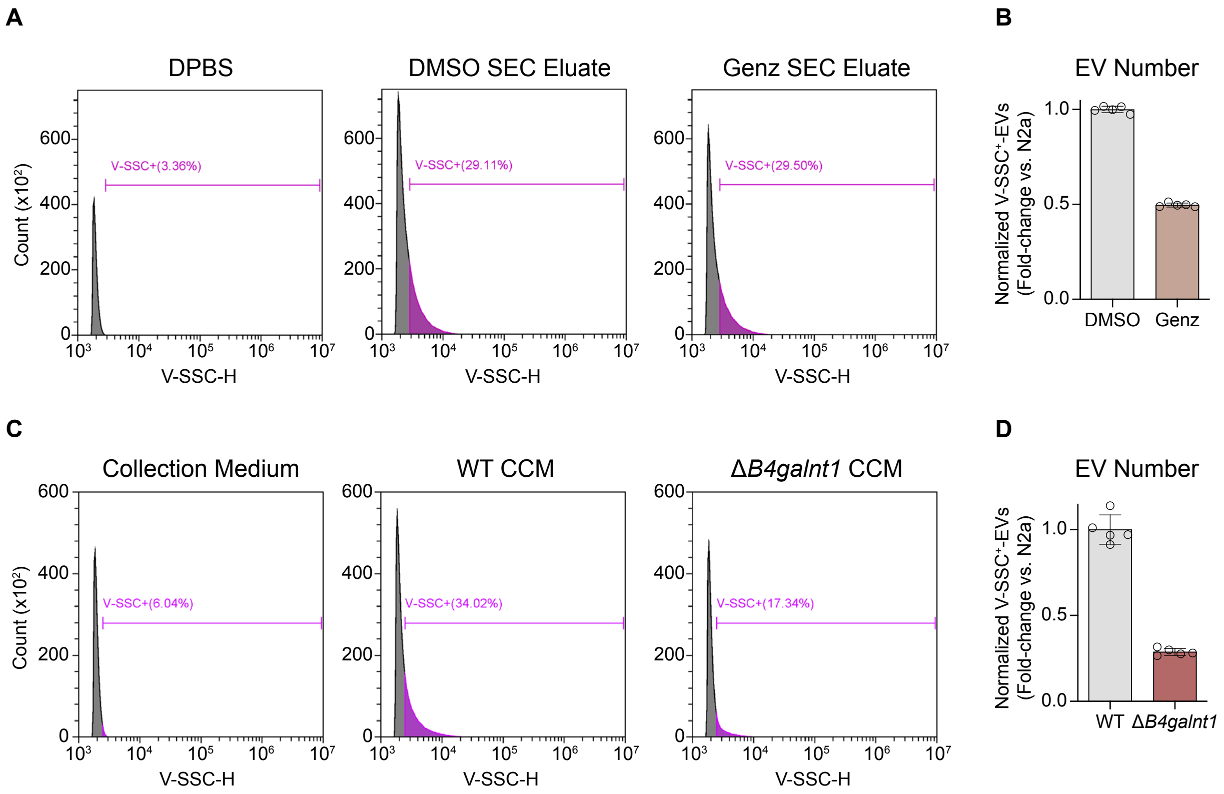
A
DPBS
DMSO SEC Eluate
Genz SEC Eluate
V-SSC+(3.36%)
V-SSC+(29.11%)
V-SSC+(29.50%)
Count (x10²)
V-SSC-H
B
EV Number
Normalized V-SSC⁺-EVs (Fold-change vs. N2a)
DMSO
Genz
C
Collection Medium
WT CCM
ΔB4galnt1 CCM
V-SSC+(6.04%)
V-SSC+(34.02%)
V-SSC+(17.34%)
Count (x10²)
V-SSC-H
D
EV Number
Normalized V-SSC⁺-EVs (Fold-change vs. N2a)
WT
ΔB4galnt1

Fig. S4. Quantification of unlabelled EVs from Genz-123346-treated and Δ*B4galnt1* N2a cells.

Gangliosides were depleted from N2a cells by treatment with Genz-123356 or *B4galnt1* knockout. Unlabelled EVs were analyzed following SEC purification or directly from the CCM by micro-flow cytometry (CytoFLEX).

(**A**) Representative histograms of violet side scatter (V-SSC) intensity of DPBS (left), SEC-isolated EVs from DMSO-treated (middle) and Genz-123346-treated N2a cells (right). The horizontal line shows the gate used to quantify EVs.

(**B**) Quantification of the number of V-SSC^{+}-EVs secreted by DMSO- or Genz-123346-treated N2a cells normalized to total cellular protein content (n=5 technical replicates).

(**C**) Representative histograms of V-SSC intensity of collection medium (left), CCM from WT N2a cells (middle), and Δ*B4galnt1* N2a cells (right).

(**D**) Quantification of the number of V-SSC^{+} EVs secreted by WT or Δ*B4galnt1* N2a cells normalized to total cellular protein content (n=5 technical replicates).

Bars indicate means ± SD.

Figure S5

A

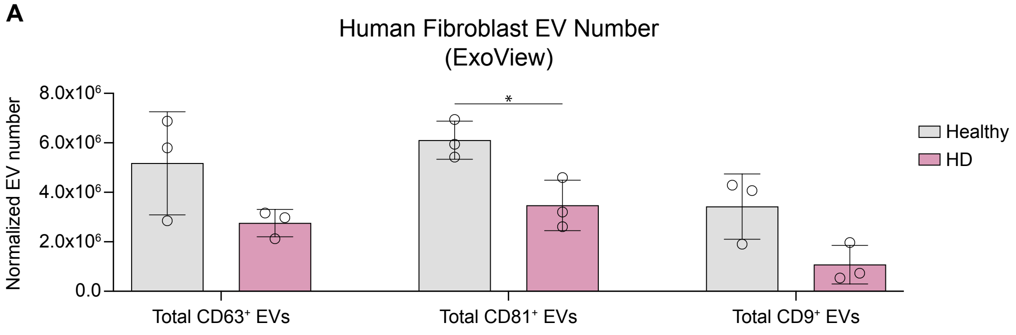

B

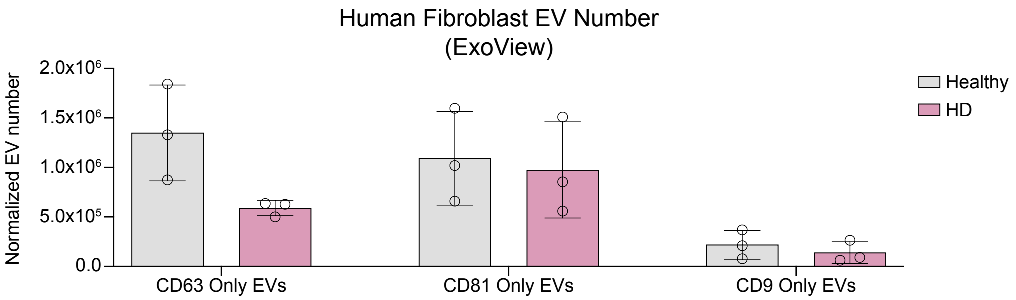

C

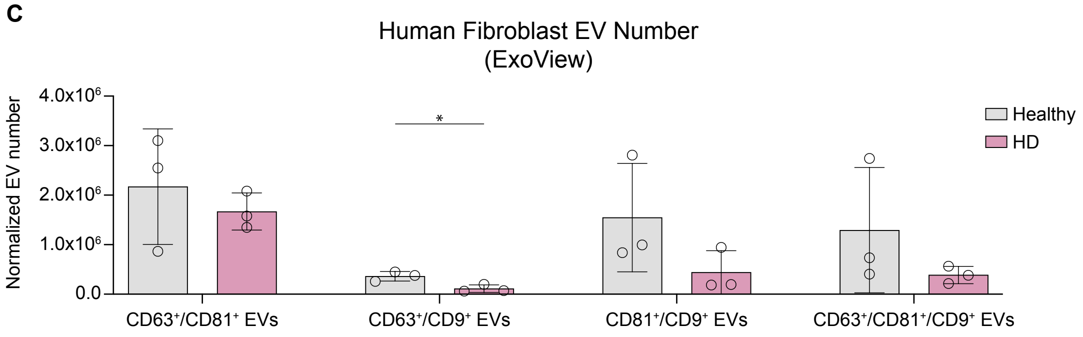

Fig. S5. Tetraspanin profile of human fibroblast EVs detected by ExoView.

EVs were collected from 3 healthy and 3 HD fibroblast cell lines. Tetraspanins were profiled by ExoView.

(**A**) Number of EVs bearing CD63, CD81 or CD9 normalized to total cellular protein content.

(**B**) Number of EVs bearing only one tetraspanin, either CD63, CD81, or CD9, normalized to total cellular protein content.

(**C**) Number of EVs bearing the indicated combinations of tetraspanins, normalized to total cellular protein content.

Bars are mean values ± SD. $^*p<0.05$, by two-tailed unpaired *t*-test.

Figure S6

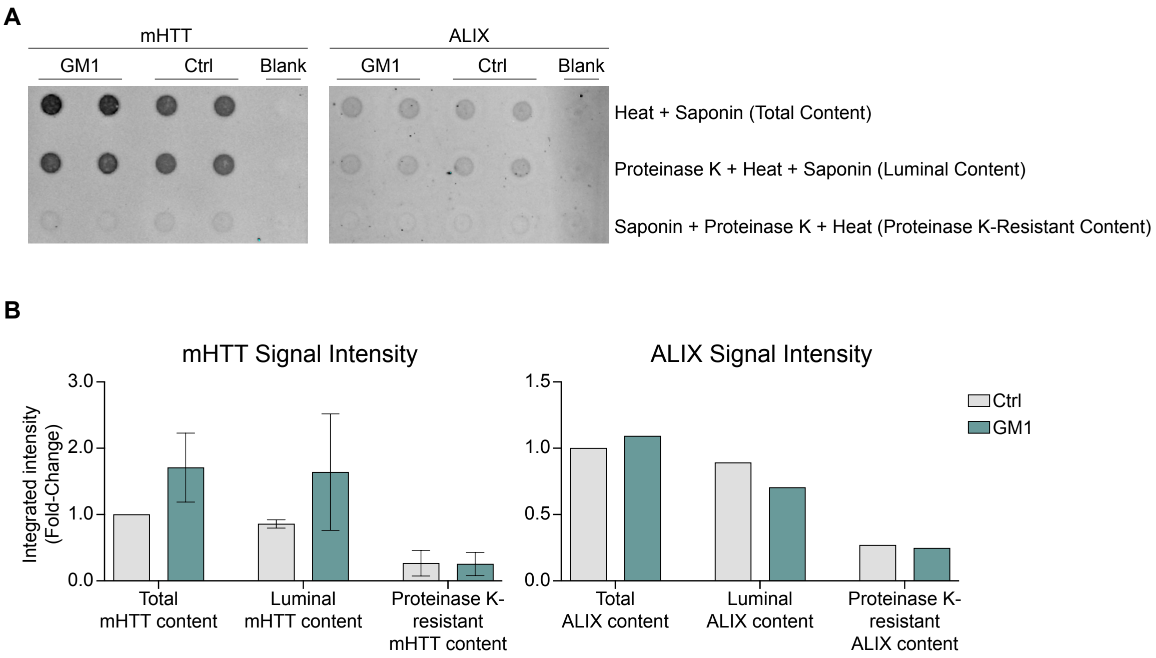
A
mHTT
ALIX
GM1
Ctrl
Blank
Heat + Saponin (Total Content)
Proteinase K + Heat + Saponin (Luminal Content)
Saponin + Proteinase K + Heat (Proteinase K-Resistant Content)
B
mHTT Signal Intensity
ALIX Signal Intensity
Integrated intensity (Fold-Change)
3.0
2.0
1.0
0.0
1.5
1.0
0.5
0.0
Total mHTT content
Luminal mHTT content
Proteinase K-resistant mHTT content
Total ALIX content
Luminal ALIX content
Proteinase K-resistant ALIX content
Ctrl
GM1

Fig. S6. The majority of mHTT associated with EVs is in the EV lumen.

EVs from N2a 97Q cells were treated with GM1 for 6 h and collected in phenol red-free, serum-free medium supplemented with N-2 for 16 h prior to isolation by SEC. EVs from control and GM1-treated cells were subjected to proteinase K digestion (0.02µg/µg of EVs) before or after EV membrane permeabilization with 0.1% saponin. In the absence of prior saponin permeabilization, proteinase K only digests proteins on the membrane and not in the lumen.

(**A**) Representative dot blot showing the effects of proteinase K on mHTT and Alix (a luminal protein) in EVs treated as indicated. Alix was used as a control for proteinase K digestion. The blank is PBS, the EV resuspension buffer.

(**B**) Densitometric analysis for mHTT (left) and Alix (right) in EVs, expressed as fold-change of undigested (heat + saponin group) untreated control (n=2 experiments for mHTT, 1 for Alix). Bars are means ± SD.

Table S1 - List of antibodies used

Antibody	Host	Catalog no.	Source	Dilution	Application	RRID
**A1P1 (ALIX)**	Mouse	611621	BD Biosciences	1:250	Western Blotting and Proteinase K Protection Assay	AB_2236941
**Calnexin**	Rabbit	ADI-SPA-860	Enzo	1:2000	Western Blotting	AB_10616095
**CD9**	Rabbit	ab92726	Abcam	1:2000	Western Blotting	AB_10561589
**Flotillin-1**	Mouse	610820	BD Biosciences	1:1000	Western Blotting	AB_398139
**GFP**	Rabbit	2956	Cell Signaling Technology	1:2000	Western Blotting	AB_1196615
**GFP**	Mouse	sc-9996	Santa Cruz	1:500	Western Blotting	AB_627695
**Huntingtin N17**	Rabbit	-	Dr. Ray Truant, McMaster University	1:5000-1:10000	Western Blotting and Proteinase K Protection Assay	-
**Huntingtin**	Mouse	MAB2166	Millipore Sigma	1:1000	Western Blotting	AB_11213141
**Tubulin**	Rabbit	2125	Cell Signaling Technology	1:1000	Western Blotting and Dot Blotting	AB_2619646
**TSG101**	Rabbit	ab125011	Abcam	1:1000	Western Blotting	AB_10974262
**α-synuclein**	Rabbit	ab19903	Abcam	1:1000	Western Blotting	AB_445200
